# Supplementary material for: General practitioners providing non-urgent care in emergency department: a natural experiment
Source: BMJ Open. 2018 May 10;8(5):e019736. doi: 10.1136/bmjopen-2017-019736 (PMC5950695; doi:10.1136/bmjopen-2017-019736)

# Contents

**ONLINE ONLY TABLES** .......... 1

**eTable 1: Any Intervention** .......... 1

**eTable 2: Any Investigation** .......... 3

**eTable 3: Patients' disposal method** .......... 4

**ONLINE ONLY FIGURES** .......... 5

**eFigure 1: Process outcomes, sensitivity analyses** .......... 5

**eFigure 2: A&E clinical indicators, sensitivity analyses** .......... 6

# ONLINE ONLY TABLES

## eTable 1: Any Intervention

| | GP in ED | | Regular Emergency Physician | |
|---|---|---|---|---|
| | No. | Col % | No. | Col % |
| Ametop | 2 | 0.0 | 2 | 0.0 |
| Arthropad | 0 | 0.0 | 4 | 0.0 |
| Backslab POP | 0 | 0.0 | 12 | 0.1 |
| Bandage/Support | 3 | 0.0 | 43 | 0.3 |
| Burns review | 0 | 0.0 | 2 | 0.0 |
| Chloramphenicol eye drops | 16 | 0.2 | 40 | 0.2 |
| Crutches | 9 | 0.1 | 141 | 0.8 |
| Digital nerve block | 0 | 0.0 | 1 | 0.0 |
| Digital splint | 0 | 0.0 | 2 | 0.0 |
| Dressing | 33 | 0.3 | 350 | 2.1 |
| Dressing/wound review | 6 | 0.1 | 12 | 0.1 |
| Eye irrigation | 2 | 0.0 | 16 | 0.1 |
| Falls prevention | 0 | 0.0 | 1 | 0.0 |
| Gastrostomy Tube Replacement | 0 | 0.0 | 2 | 0.0 |
| Guidance - verbal | 4241 | 39.9 | 6678 | 39.3 |
| Guidance - written | 96 | 0.9 | 551 | 3.2 |
| Guidance / Advice only | 23 | 0.2 | 109 | 0.6 |
| Infusion fluids | 0 | 0.0 | 4 | 0.0 |
| Intravenous cannula | 68 | 0.6 | 133 | 0.8 |
| Knee splint | 1 | 0.0 | 4 | 0.0 |
| Lavage/emesis/charcoal/eye irrigation | 0 | 0.0 | 9 | 0.1 |
| Manipulation | 1 | 0.0 | 0 | 0.0 |
| Medication - ear drops | 10 | 0.1 | 7 | 0.0 |
| Medication - eye drops | 12 | 0.1 | 73 | 0.4 |
| Medication - intra-nasal | 2 | 0.0 | 2 | 0.0 |
| Medication - oral | 382 | 3.6 | 374 | 2.2 |
| Medication - per rectum | 29 | 0.3 | 41 | 0.2 |
| Medication - topical skin cream | 22 | 0.2 | 7 | 0.0 |
| Medication administered | 1 | 0.0 | 4 | 0.0 |
| NSAID | 184 | 1.7 | 223 | 1.3 |
| Nasogastric Tube Replacement | 0 | 0.0 | 3 | 0.0 |
| Neighbour strapping | 9 | 0.1 | 129 | 0.8 |
| None (consider guidance/advice option) | 11 | 0.1 | 37 | 0.2 |
| Observation | 3673 | 34.6 | 4534 | 26.7 |
| Observation/electrocardiogram/pulse ox/head injury/trends | 115 | 1.1 | 684 | 4.0 |
| Oral Analgesia | 476 | 4.5 | 722 | 4.2 |
| Oral Rehydration Therapy | 15 | 0.1 | 20 | 0.1 |
| Oral antibiotics | 408 | 3.8 | 376 | 2.2 |
| Other (consider alternatives) | 0 | 0.0 | 12 | 0.1 |

| | **GP in ED** | | **Regular Emergency Physician** | |
|---|---|---|---|---|
| Prescription/medicines prepared to take away | 663 | 6.2 | 690 | 4.1 |
| Recording vital signs | 0 | 0.0 | 10 | 0.1 |
| Shortwave diathermy | 1 | 0.0 | 0 | 0.0 |
| Sltra sound treatment | 1 | 0.0 | 0 | 0.0 |
| Sling/collar cuff/broad arm sling | 7 | 0.1 | 90 | 0.5 |
| Splint | 3 | 0.0 | 11 | 0.1 |
| Tetanus | 2 | 0.0 | 3 | 0.0 |
| Tetanus - human immunoglobulin | 0 | 0.0 | 1 | 0.0 |
| Tetanus - immune | 0 | 0.0 | 1 | 0.0 |
| Wound cleaning | 34 | 0.3 | 388 | 2.3 |
| Wound closure (excluding sutures) | 0 | 0.0 | 8 | 0.0 |
| Wound irrigation | 1 | 0.0 | 56 | 0.3 |
| Wrist splint | 2 | 0.0 | 12 | 0.1 |
| combined tetanus/diphtheria course | 13 | 0.1 | 50 | 0.3 |
| dressing minor wound/burn/eye | 4 | 0.0 | 20 | 0.1 |
| entonox | 0 | 0.0 | 6 | 0.0 |
| gait re-education | 0 | 0.0 | 1 | 0.0 |
| local anaesthetic | 2 | 0.0 | 10 | 0.1 |
| removal Plaster of Paris | 0 | 0.0 | 1 | 0.0 |
| removal of sutures/clips | 1 | 0.0 | 13 | 0.1 |
| steristrips | 14 | 0.1 | 136 | 0.8 |
| strapping | 1 | 0.0 | 0 | 0.0 |
| tetanus toxoid booster | 0 | 0.0 | 3 | 0.0 |
| wound glue | 18 | 0.2 | 126 | 0.7 |
| Total | 10617 | 100.0 | 17000 | 100.0 |

eTable 2: Any Investigation

| | GP in ED | | Regular Emergency Physician | |
|---|---|---|---|---|
| | No. | Col % | No. | Col % |
| Arterial/capillary blood gas | 12 | 0.1 | 132 | 0.6 |
| BM Stix | 1243 | 14.9 | 1259 | 6.2 |
| Bacteriology | 5 | 0.1 | 18 | 0.1 |
| Biochemistry | 19 | 0.2 | 119 | 0.6 |
| Blood culture | 10 | 0.1 | 86 | 0.4 |
| CT (excludes GU contrast exam/tomography) | 0 | 0.0 | 1 | 0.0 |
| Cardiac enzymes | 87 | 1.0 | 147 | 0.7 |
| Clotting studies | 106 | 1.3 | 594 | 2.9 |
| Cross match blood/group and save serum for later cross match | 4 | 0.0 | 38 | 0.2 |
| D-Dimer | 90 | 1.1 | 141 | 0.7 |
| Dental Investigation | 0 | 0.0 | 1 | 0.0 |
| ESR | 22 | 0.3 | 28 | 0.1 |
| Electrocardiogram | 748 | 9.0 | 788 | 3.9 |
| Full blood count | 583 | 7.0 | 2148 | 10.5 |
| Glucose | 63 | 0.8 | 291 | 1.4 |
| Group & Save | 24 | 0.3 | 206 | 1.0 |
| Haematology | 339 | 4.1 | 1644 | 8.0 |
| Histology | 0 | 0.0 | 3 | 0.0 |
| INR | 30 | 0.4 | 168 | 0.8 |
| Immunology | 2 | 0.0 | 1 | 0.0 |
| Liver Function Test | 490 | 5.9 | 1999 | 9.8 |
| None | 2855 | 34.2 | 4404 | 21.6 |
| Orthoptic tests and computerised visual fields | 0 | 0.0 | 14 | 0.1 |
| Other | 5 | 0.1 | 257 | 1.3 |
| Other Biochemistry | 0 | 0.0 | 1 | 0.0 |
| Pregnancy test | 165 | 2.0 | 413 | 2.0 |
| Refraction | 0 | 0.0 | 14 | 0.1 |
| Serology | 1 | 0.0 | 7 | 0.0 |
| Tetanus | 2 | 0.0 | 0 | 0.0 |
| Toxicology | 0 | 0.0 | 1 | 0.0 |
| Urea & Electrolytes | 574 | 6.9 | 2161 | 10.6 |
| Urinalysis | 414 | 5.0 | 890 | 4.4 |
| Urine Culture | 16 | 0.2 | 27 | 0.1 |
| Wound Culture | 0 | 0.0 | 5 | 0.0 |
| X-ray plain film | 433 | 5.2 | 2418 | 11.8 |
| Total | 8342 | 100.0 | 20424 | 100.0 |

eTable 3: Patients' disposal method

| | GP in ED | | Regular Emergency Physician | |
|---|---|---|---|---|
| | No. | Col % | No. | Col % |
| Clinic 9 | 0 | 0.0 | 916 | 8.4 |
| Admitted to bed/became a LODGED PATIENT of the same Health Care Provider | 301 | 5.5 | 2152 | 19.8 |
| Died in Department | 0 | 0.0 | 2 | 0.0 |
| Discharged - did not require any follow up treatment | 2370 | 43.7 | 4127 | 38.0 |
| Discharged - follow up treatment to be provided by GP | 1545 | 28.5 | 1733 | 16.0 |
| Left Department before being treated | 121 | 2.2 | 439 | 4.0 |
| Left Department having refused treatment | 4 | 0.1 | 41 | 0.4 |
| Other | 11 | 0.2 | 68 | 0.6 |
| Referred to A&E Clinic | 804 | 14.8 | 524 | 4.8 |
| Referred to Fracture Clinic | 22 | 0.4 | 301 | 2.8 |
| Referred to other Out-Patient Clinic | 198 | 3.6 | 374 | 3.4 |
| Referred to other health care professional | 46 | 0.8 | 161 | 1.5 |
| Transferred to other Health Care Provider | 4 | 0.1 | 14 | 0.1 |
| Total | 5426 | 100.0 | 10852 | 100.0 |

# ONLINE ONLY FIGURES

## eFigure 1: Process outcomes, sensitivity analyses

| Outcome | % GP−in−ED (n=5,426) | % Regular ED Care (n=10,852) | RR | 95%−CI |
|---|---|---|---|---|
| **Any admission** | | | | |
| Nearest Neighbour, without replacement | 5.5 | 20.1 | 0.28 | [0.25; 0.31] |
| Nearest Neighbour, with replacement | 5.5 | 19.3 | 0.29 | [0.26; 0.32] |
| Exact algorithm, without replacement | 5.5 | 20.6 | 0.27 | [0.24; 0.30] |
| Exact algorithm, with replacement | 5.5 | 20.6 | 0.27 | [0.24; 0.30] |
| **Referrals to other specialists** | | | | |
| Nearest Neighbour, without replacement | 1.3 | 4.3 | 0.31 | [0.24; 0.40] |
| Nearest Neighbour, with replacement | 1.3 | 4.3 | 0.31 | [0.24; 0.39] |
| Exact algorithm, without replacement | 1.3 | 4.3 | 0.31 | [0.25; 0.39] |
| Exact algorithm, with replacement | 1.3 | 4.3 | 0.31 | [0.25; 0.39] |
| **Any radiological investigation** | | | | |
| Nearest Neighbour, without replacement | 7.2 | 18.9 | 0.38 | [0.34; 0.42] |
| Nearest Neighbour, with replacement | 7.2 | 18.3 | 0.39 | [0.35; 0.43] |
| Exact algorithm, without replacement | 7.2 | 19.0 | 0.38 | [0.34; 0.42] |
| Exact algorithm, with replacement | 7.2 | 19.0 | 0.38 | [0.34; 0.42] |
| **Any blood investigation** | | | | |
| Nearest Neighbour, without replacement | 12.1 | 21.5 | 0.57 | [0.52; 0.61] |
| Nearest Neighbour, with replacement | 12.1 | 21.1 | 0.58 | [0.53; 0.62] |
| Exact algorithm, without replacement | 12.1 | 19.8 | 0.61 | [0.57; 0.66] |
| Exact algorithm, with replacement | 12.1 | 19.8 | 0.61 | [0.57; 0.66] |
| **Any investigation** | | | | |
| Nearest Neighbour, without replacement | 45.9 | 49.3 | 0.93 | [0.90; 0.96] |
| Nearest Neighbour, with replacement | 45.9 | 49.6 | 0.92 | [0.89; 0.96] |
| Exact algorithm, without replacement | 45.8 | 47.1 | 0.97 | [0.94; 1.00] |
| Exact algorithm, with replacement | 45.8 | 47.1 | 0.97 | [0.94; 1.00] |
| **Any intervention (including prescription)** | | | | |
| Nearest Neighbour, without replacement | 35.6 | 27.5 | 1.29 | [1.23; 1.35] |
| Nearest Neighbour, with replacement | 35.6 | 28.2 | 1.26 | [1.20; 1.32] |
| Exact algorithm, without replacement | 35.5 | 28.6 | 1.24 | [1.20; 1.29] |
| Exact algorithm, with replacement | 35.5 | 28.6 | 1.24 | [1.20; 1.29] |
| **Follow−up with primary care physician** | | | | |
| Nearest Neighbour, without replacement | 28.5 | 16.0 | 1.78 | [1.67; 1.89] |
| Nearest Neighbour, with replacement | 28.5 | 16.7 | 1.70 | [1.60; 1.81] |
| Exact algorithm, without replacement | 28.6 | 14.7 | 1.94 | [1.85; 2.03] |
| Exact algorithm, with replacement | 28.6 | 14.7 | 1.94 | [1.85; 2.03] |
| **Referrals to out−patient / other A&E clinic** | | | | |
| Nearest Neighbour, without replacement | 18.5 | 8.1 | 2.29 | [2.10; 2.49] |
| Nearest Neighbour, with replacement | 18.5 | 8.1 | 2.27 | [2.08; 2.47] |
| Exact algorithm, without replacement | 18.6 | 7.1 | 2.61 | [2.45; 2.77] |
| Exact algorithm, with replacement | 18.6 | 7.1 | 2.61 | [2.45; 2.77] |

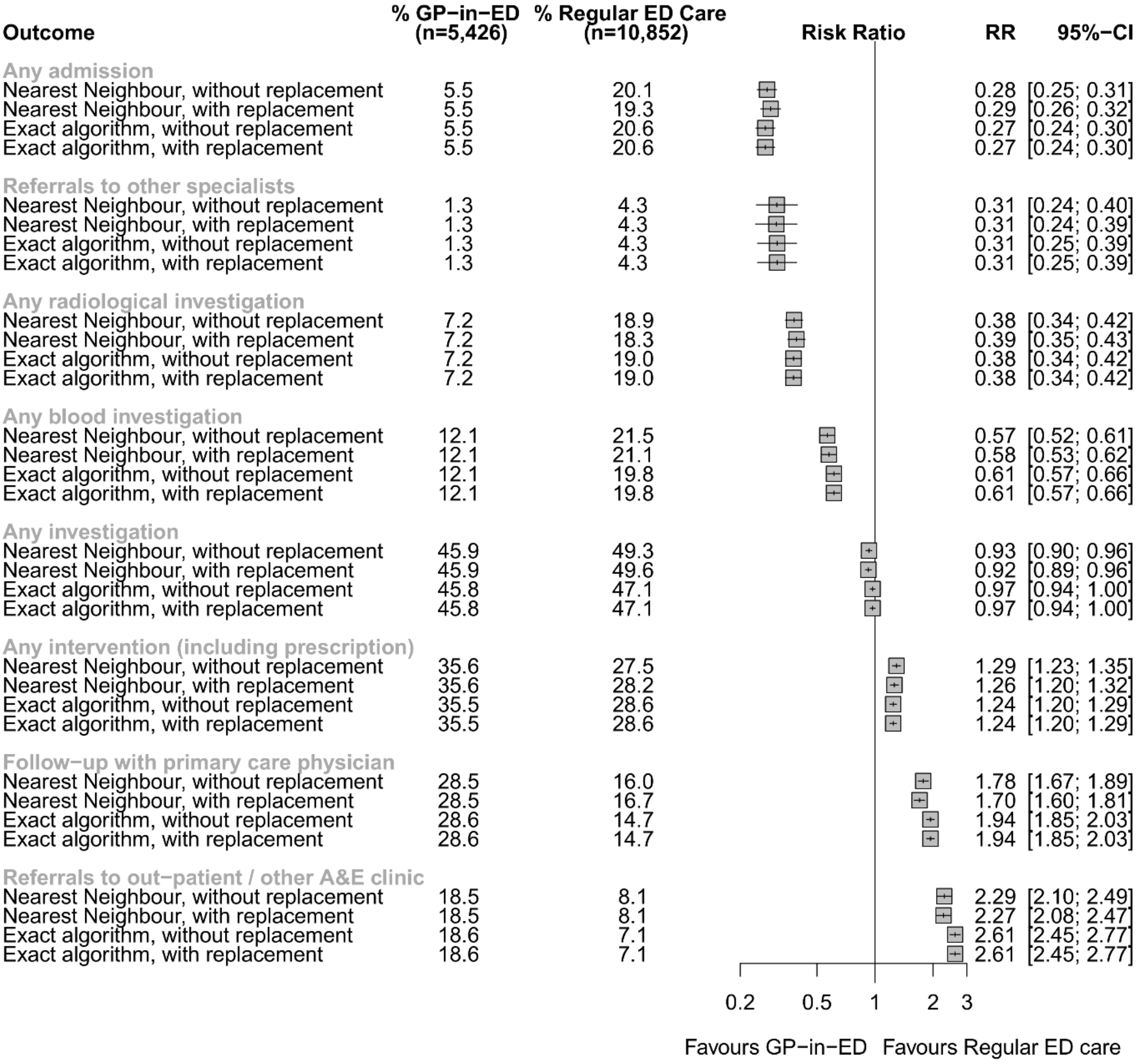

eFigure 2: A&E clinical indicators, sensitivity analyses

| Outcome | % GP−in−ED (n=5,426) | % Regular ED Care (n=10,852) | Risk Ratio | RR | 95%−CI |
|---|---|---|---|---|---|
| **Spent for 4−hour plus** | | | | | |
| Nearest Neighbour, without replacement | 1.9 | 5.3 | | 0.37 | [0.30; 0.45] |
| Nearest Neighbour, with replacement | 1.9 | 6.0 | | 0.32 | [0.26; 0.39] |
| Exact algorithm, without replacement | 2.0 | 4.9 | | 0.40 | [0.33; 0.48] |
| Exact algorithm, with replacement | 2.0 | 4.9 | | 0.40 | [0.33; 0.48] |
| **Left without being seen** | | | | | |
| Nearest Neighbour, without replacement | 2.2 | 3.9 | | 0.57 | [0.46; 0.69] |
| Nearest Neighbour, with replacement | 2.2 | 4.0 | | 0.55 | [0.45; 0.68] |
| Exact algorithm, without replacement | 2.3 | 3.4 | | 0.66 | [0.55; 0.80] |
| Exact algorithm, with replacement | 2.3 | 3.4 | | 0.66 | [0.55; 0.80] |
| **Seven day re−attendance** | | | | | |
| Nearest Neighbour, without replacement | 5.5 | 5.7 | | 0.96 | [0.84; 1.09] |
| Nearest Neighbour, with replacement | 5.5 | 6.0 | | 0.91 | [0.80; 1.04] |
| Exact algorithm, without replacement | 5.5 | 6.3 | | 0.88 | [0.78; 0.98] |
| Exact algorithm, with replacement | 5.5 | 6.3 | | 0.88 | [0.78; 0.98] |

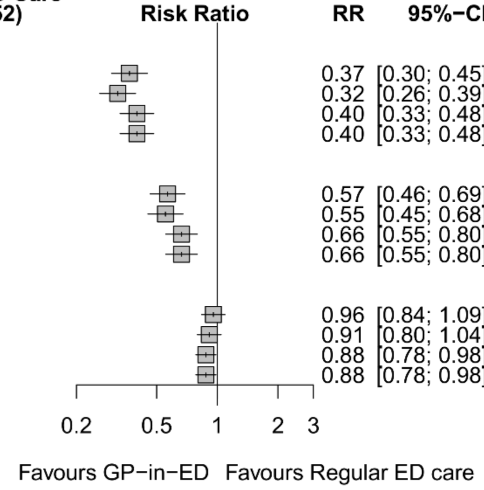

Supplement: Supplementary file 1 [file bmjopen-2017-019736supp001.pdf]
