# Supplementary material for: General practitioners providing non-urgent care in emergency department: a natural experiment
Source: BMJ Open. 2018 May 10;8(5):e019736. doi: 10.1136/bmjopen-2017-019736 (PMC5950695; doi:10.1136/bmjopen-2017-019736)
Supplement: Author's manuscript [file bmjopen-2017-019736.draft_revisions.pdf]

BMJ Open is committed to open peer review. As part of this commitment we make the peer review history of every article we publish publicly available.

When an article is published we post the peer reviewers' comments and the authors' responses online. We also post the versions of the paper that were used during peer review. These are the versions that the peer review comments apply to.

The versions of the paper that follow are the versions that were submitted during the peer review process. They are not the versions of record or the final published versions. They should not be cited or distributed as the published version of this manuscript.

BMJ Open is an open access journal and the full, final, typeset and author-corrected version of record of the manuscript is available on our site with no access controls, subscription charges or pay-per-view fees (http://bmjopen.bmj.com).

If you have any questions on BMJ Open's open peer review process please email info.bmjopen@bmj.com

BMJ Open

General practitioners providing non-urgent care in emergency department: evaluation of a natural experiment

Journal:	*BMJ Open*
Manuscript ID	bmjopen-2017-019736
Article Type:	Research
Date Submitted by the Author:	25-Sep-2017
Complete List of Authors:	Uthman, Olalekan; Warwick Medical School,<br>Walker, Clare; University of Warwick Warwick Medical School<br>Lahiri, Sudakshina; University of Warwick, Institute of Digital Healthcare, WMG<br>Jenkinson, David; University of Warwick, Warwick Medical School<br>Adekanmbi, Victor; University of Warwick Warwick Medical School<br>Robertson, Wendy; University of Warwick Warwick Medical School<br>Clarke, Aileen; University of Warwick, Division of Health Sciences
**Primary Subject Heading**:	Emergency medicine
Secondary Subject Heading:	General practice / Family practice
Keywords:	PRIMARY CARE, ACCIDENT & EMERGENCY MEDICINE, HEALTH SERVICES ADMINISTRATION & MANAGEMENT, Organisation of health services < HEALTH SERVICES ADMINISTRATION & MANAGEMENT

SCHOLARONE™
Manuscripts

General practitioners providing non-urgent care in emergency department: evaluation of a natural experiment

Olalekan A. Uthman[1], Clare Walker[1], Sudakshina Lahiri[2], David Jenkinson[1], Victor Adekanmbi[1], Wendy Robertson[1], Aileen Clarke[1]

[1]Division of Health Sciences, Warwick Medical School, The University of Warwick, Coventry, CV4 7AL, United Kingdom;

[2] Institute of Digital Healthcare, WMG, The University of Warwick, Coventry

Corresponding author: Prof. Aileen Clarke, Division of Health Sciences, Warwick Medical School, The University of Warwick, Coventry, CV4 7AL, United Kingdom.

Aileen.Clarke@warwick.ac.uk.

For peer review only - http://bmjopen.bmj.com/site/about/guidelines.xhtml

ABSTRACT

Objective: To examine whether care provided by general practitioners (GPs) to non-urgent patients in the emergency department (ED) differs significantly from care provided by usual accident and emergency (A&E) staff in terms of process outcomes and A&E clinical quality indicators.

Design: Propensity score matched cohort study

Setting: The University Hospitals Coventry and Warwickshire NHS Trust (UHCW)

Participants: Non-Urgent Attendances (self-reporting minor cases), unplanned visits to the hospital that appeared as: either "self-referrals" or referrals made by "family or friends"; i.e. those presenting with minor illness.

Main outcomes: Process outcomes (any investigation, any blood investigation, any radiological investigation, any intervention, admission and referrals) and A&E Clinical indicators (spent 4 hours plus, left without being seen, and seven-day re-attendance).

Results: A total of 5,426 patients seen by GPs in A&E were matched with 10,852 patients seen by emergency physicians (ratio 1:2). Compared with standard care in A&E standard care in A&E, GPs in A&E significantly: referred fewer patients to other specialists, admitted fewer patients, ordered fewer radiological investigations, ordered fewer blood tests, and ordered fewer investigations. However, they intervened more, offered more primary care follow up, and referred more patients to out-patient and other A&E clinics. Patients seen by GPs in A&E were on average less likely to spend four hours plus in A&E, and were less likely to be admitted compared with standard care in A&E. There was no difference in re-attendance after seven days.

Conclusion: GPs in A&E tended to manage self-reporting minor cases with fewer resources than standard care in A&E, without increasing re-attendance rates.

For peer review only - http://bmjopen.bmj.com/site/about/guidelines.xhtml

Strengths and limitations of this study

- Findings from this study are among the first to provide empirical evidence that GPs in A& E are offering better continuity of care (perhaps more comprehensive care)
- Our results have important clinical implications as they inform optimal care for non-urgent care patients in A&E
- The main limitation of the study is the lack of random allocation and as such our findings may be prone to selection bias.
- We do not know the eventual health outcome, although re-attendance data are reassuring on this front.

For peer review only

For peer review only - http://bmjopen.bmj.com/site/about/guidelines.xhtml

BACKGROUND

Accident and Emergency departments (A&E) in the United Kingdom (UK) are facing a crisis in terms of overcrowding with long waiting times. Numerous factors have been suggested for this overcrowding in the use of A&E services, including ‘inappropriate’ use by patients with non-urgent conditions. A wide range (between 6.7-89%) of A&E visits has been classified as non-urgent depending on definition; and it is thought that many patients could have their care needs met in non-hospital including in General Practitioner (GP) settings [1-5]. Inappropriate use is thought to compromise A&E services for true emergencies [6 7]. And, it has been suggested that non-urgent cases might be managed hastily or may not be linked in with the comprehensive and continuous care available in the primary care setting, thus leading to sub-optimal care [1].

Reducing inappropriate use of A&E has been an important area for policy makers for a long time[8], and experts have recently recommended that “All hospital emergency departments in the United Kingdom should include a primary care out-of-hours facility to help ease the ongoing pressure on the system” [9]. The “introduction of GPs may provide more comprehensive and cost- and resource-effective care for patients with non-urgent problems in the ED” it is thought [10]. It has also been suggested that GPs in A&E could reduce waiting times by seeing non-urgent patients promptly and freeing regular A&E physicians to see more urgent cases [11].

For peer review only

For peer review only - http://bmjopen.bmj.com/site/about/guidelines.xhtml

Existing studies that have examined the effects of introducing GPs in A&E to provide care for non-urgent cases compared with standard care in A&E [10 12-14]. However, these studies have focused on process outcomes only. To our knowledge, no studies have examined the impact of GP in A&E on accident and emergency (A&E) clinical indicators. The present evaluation deals with a critical research gap highlighted by a previous Cochrane Review[10], described as the need for further studies of the effects of GP in A&E "on wait times, adverse effects, mortality and patient outcomes". The main purpose of this study was therefore to examine whether care provided to non-urgent patients by GPs in the A&E differed significantly from care by usual A&E staff in terms of process outcomes and A&E clinical quality indicators.

METHODS

Study design, setting and source of data

We used anonymized individual level acute care activity data from the University Hospitals Coventry and Warwickshire NHS Trust (UHCW) between May 2015 and March 2016. We used the Urgent Care Project Board definition[15] to identify non-urgent attendances (self-reporting minor cases). These are unplanned visits to the A&E defined as either "self-referrals" or referrals made by "family or friends" and associated with Healthcare Resource Groups (HRG) codes which signify minor presentations; i.e. those presenting with minor illness with a National Early Warning Score (NEWS) ≤ 1. The three relevant HRG codes are: VB08Z (Emergency Medicine, Category 2 investigation with Category 1 Treatment), VB09Z (Emergency Medicine, Category 1 investigation with Category 1 - 2 Treatment) and VB11Z (Emergency Medicine, No investigation with No Significant Treatment).

For peer review only - http://bmjopen.bmj.com/site/about/guidelines.xhtml

Main exposure variable: GP in A&E scheme

The exposure of interest was whether the patients were seen by an emergency physician or by a GP in A&E. The GP in A&E scheme was set up by the Coventry and Rugby Clinical Commissioning Group (CRCCG) as part of the Prime Minister's Access Fund (PMAF) initiative.

The patients arrived at the A&E reception on foot, into the paediatric A&E or into the ambulance entrance, and were registered onto the Trust's clinical A&E system (iPM). Triage was undertaken initially by the A&E triage nurse and by either an advanced nurse practitioner (ANP) or a senior nurse, who also undertook an initial visual assessment of the patient. Patients suitable for GP in A&E were identified and those deemed unsuitable were returned to existing A&E clinical pathways. Staff in the GP in A&E service also actively identified cases based on observation of patients in A&E, and/or based on information recorded in patient registration notes. Patients were not randomised. Once the decision to transfer to GP in A&E was made, the patient was moved onto the iPM by a receptionist and into the GP in A&E clinic. Patients suitable for the service were then diagnosed and treated by a GP in A&E. The patient's GP practice notes (accessed via the primary care shared care record) were available during the consultation. Patients who needed further tests or admission to hospital were referred to UHCW existing hospital services. Onward referral post discharge to a registered GP was via access to the primary care shared care record. The GP in A&E scheme operated seven days a week, for 12 hours per day

Outcomes and covariates:

The outcomes of interest were:

For peer review only - http://bmjopen.bmj.com/site/about/guidelines.xhtml

1. Process outcomes: any investigations, any blood investigations, any radiological investigations, any intervention (including prescriptions) and admissions, discharges, referrals to outpatient clinics or other A&E clinics, referrals to other specialists, and follow-up treatment to be provided by GP in primary care setting.
2. A&E Clinical indicators: percentage of patients who spent 4 hours plus, who left without being seen, and seven-day re-attendance rates.

Confounding variables included in the analyses were patients' age, sex, ethnicity, number of procedures, distance to UHCW (partial post code), and number of presenting symptoms.

Statistical analyses

We examined the baseline characteristics of the patient cohort and estimated standardized differences for all variables before and after matching. A standardized difference of 10% or more is suggestive of imbalance [15]. We used propensity score[16] methods to account for all measured differences in baseline characteristics between patients seen by GP in A&E and standard care in A&E. The propensity score approach was used to control for all observed confounding factors that might influence assignment and outcome. We constructed a sample of patients balanced on covariates and risk factors (such as age, sex, ethnicity, number of procedures and number of presentations). We constructed the propensity scores using a logistic regression. We then matched each patient seen by GP in A&E to another similar standard care in A&E patients with the closest propensity score on a ratio of 1:2 using a nearest neighbour algorithm with no replacement. We calculated the average treatment effect on treated patients, which is a measure of the impact of GP in A&E on process outcomes and A&E clinical indicators. All data were analysed using R statistical

For peer review only - http://bmjopen.bmj.com/site/about/guidelines.xhtml

software version 3.4.0. The null hypothesis was tested against a two-sided alternative hypothesis at significance level of 5%.

Patient involvement

The Collaborations for Leadership in Applied Health Research and Care (CLAHRC) West Midlands Patient and Public Involvement (PPI) advisors reviewed the study on a number of occasions. They were involved with the design of the research question and gave feedback at our advisory committee meeting. The research question was generated by the Coventry Local Authority who were the funders of the study. The results were discussed with PPI advisors and local authorities at a large stakeholder event. The results have been disseminated via our local authorities and shared through patient and public involvement initiatives.

For peer review only

For peer review only - http://bmjopen.bmj.com/site/about/guidelines.xhtml

RESULTS

Patients and Characteristics

A total of 170,154 patients attended UHCW A&E between May 2015 and March 2016 (Fig 1a). Our primary cohort consisted of 120,034 self-reporting minor case attendances in A&E at UHCW. Of every 10 attendees, 7 were self-reporting minor cases during this period. Attendances were relatively even throughout the week, with the highest attendances on a Monday (approximately 16%). Attendance was greatest on each day between 9-11am (Fig 1b). Most patients (70%) were dealt with, within 3 hours of attendance at A&E (Fig 1c).

Characteristics of the patients before and after matching are summarized in Table 1 (and supplementary eTable 1, eTable 2, and eTable 3). There were important baseline differences between patients seen by GPs in A&E and those seen in the normal pathway. GPs in A&E tended to see fewer white patients (69.9% versus 80.1%), and patients who lived closer to the hospital; 43.3% of GP in A&E patients lived within a 5 miles' radius of the A&E compared to 30.1% in standard care in A&E group. We successfully matched 5,426 patients seen by GPs in A&E with 10,852 standard care in A&E patients. After matching, absolute standardized differences were less than 10% for all variables used for the propensity score matching, suggesting an adequate match.

Process outcomes

Process outcomes and A&E clinical indicators are presented in Figure 2. GPs in A&E referred fewer patients to other specialists (68/5426, 1.2%) than standard care in A&E(1378/10852,

For peer review only - http://bmjopen.bmj.com/site/about/guidelines.xhtml

12.7%, Risk Ratio (RR) 0.10, 95% CI 0.08 to 0.13). GPs in A&E admitted significantly fewer patients with self-reporting minor cases (301/5426, 5.5%) than standard care in A&E (2061/10852, 19.0%; RR 0.29, 95% CI 0.26 to 0.33). GPs in A&E were significantly less likely than standard care in A&E to order radiological investigations (RR 0.38, 95% CI 0.34 to 0.42) and blood tests (RR 0.54, 95% CI 0.50 to 0.60) for patients. Also, on average, GPs in A&E ordered significantly but slightly fewer investigations overall, than standard care in A&E (RR 0.94, 95% CI 0.91 to 0.97). GPs in A&E intervened more (RR 1.28, 95% CI 1.22 to 1.34) and referred significantly more patients to out-patients and other A&E clinic (RR 2.23, 95% CI 2.05 to 2.43) than standard care in A&E.

GPs in A&E also discharged more patients with the offer of significantly more primary care follow up than standard care in A&E(RR 1.78, 95% 1.68 to 1.89).

A&E Clinical Indicators

The proportion of patients (all self-reporting minor cases) who spent four hours plus in the A&E was statistically significantly lower for patients seen by the GPs in A&E (105/5426, 1.9%) than for those seen by standard care in A&E (608/10852, 5.6%; RR 0.35, 95% CI 0.28 to 0.42). Patients assigned to GP in A&E were less likely to leave without being seen (121/5426, 2.2%) compared with those assigned to standard care in A&E (444/10852, 4.1%; RR 0.55, 95% CI 0.45 to 0.66). The rate of seven-day re-attendance was similar among those patients seen by GP in A&E (296/5426, 5.5%) and emergency physicians (635/10852, 5.9%; RR 0.93, 95% CI 0.82 to 1.07).

DISCUSSION

For peer review only - http://bmjopen.bmj.com/site/about/guidelines.xhtml

Main findings

We found significant differences between, GPs in A&E and standard care in A&E. GPs in A&E referred fewer patients to other specialists, admitted fewer patients, ordered fewer radiological investigations, fewer blood tests, and fewer investigations overall and intervened more often. They offered more primary care follow up and referred more patients to out-patient and other A&E clinics. More importantly, on average, patients seen by GPs in A&E: were less likely to spend four hours plus in A&E and were no more likely to re-attend after seven days. The findings corroborate those of the previous three non-randomized studies[12-14] included in a Cochrane review[10]. The review involved more than 10,000 non-urgent cases from 52 A&E units, and provided evidence that GPs in A&E ordered fewer x-rays and admitted fewer patients compared with standard care in A&E. Our study also showed that GPs prescribed oral medication and to take out (TTO) medication more often, more oral antibiotics, more topical creams, and more ear drops, More TTOs may mean they are writing more repeat prescriptions or change existing prescriptions to optimise therapy. GPs prescribe less eye drops in general, slightly less rectal medication and nonsteroidal anti-inflammatory drugs (NSAIDs). Although they gave verbal guidance at similar rates as standard care in A&E.

Study limitations and strengths

The main limitation of the study is the lack of random allocation[17 18]. Our findings may be prone to selection bias since although we used a propensity score matching method to control for known baseline differences, it is possible that we have not controlled for important confounders, such as severity of presentation. We did however adjust our

For peer review only - http://bmjopen.bmj.com/site/about/guidelines.xhtml

analysis for number of presenting symptoms, number of procedures conducted and the number of tests ordered. In addition, we do not know the eventual health outcome, although re-attendance data are reassuring on this front.

Notwithstanding these limitations, these findings are among the first to provide empirical evidence about GPs in A&E and A&E clinical indicators, supporting theories that "the introduction of GPs may provide more comprehensive and cost- and resource-effective care for patients with non-urgent problems in the A&E"[10] The 2016 and 2017 "extraordinary pressures" on A&E services across the country and schemes like this one will play a part in relieving this pressure. Our findings suggest that GPs are offering better continuity of care (perhaps more comprehensive care.) We hypothesise that the fact that the GPs in A&E had access to primary care notes may be of importance here. The prescribing data we examined could mean that GPs in A&E are fulfilling requests for repeat medications more often than A&E staff with the added safety of access to the primary care record. In addition, findings suggest that GPs were making efficient use of resources by referring more non-urgent patients to ambulatory clinics such as the urgent primary care clinic in UHCW or providing secondary care outpatient referrals and avoiding hospital admission. We used data from one of the largest acute teaching hospitals in the UK. Our propensity score approach allowed us to reduce (though not eliminate) the possibility of confounding in relation to several relevant aspects of severity of presentation, as well as allowing us to balance a wide range of socio-demographic characteristics. The approach is consistent with Medical Research Council (MRC) guidelines for using natural experiments to evaluate population health interventions[19].

For peer review only - http://bmjopen.bmj.com/site/about/guidelines.xhtml

Conclusion

In this observational study of patients self-reporting with minor illness to the A&E department in one large teaching hospital, GPs in A&E made fewer referrals to other specialists, admitted fewer patients, ordered fewer radiological investigations, fewer blood tests, fewer investigations overall and intervened more often, compared to standard care in A&E. Overall they managed patients with fewer resources than standard care in A&E, without increasing re-attendance rates.

For peer review only

For peer review only - http://bmjopen.bmj.com/site/about/guidelines.xhtml

Contributors: OAU, CW, SL, DJ, WR, and AC were responsible for study concept and design. OAU and SL were responsible for acquisition of data. OAU, CW and AC drafted of the manuscript. OAU, SL, and VA performed the statistical analysis. All authors interpreted the data, critically revised the manuscript for important intellectual content and approved the final version for submission. All authors agree to be accountable for all aspects of the work. OAU and AC are guarantors.

Funding: Coventry – Rugby GP Alliance

Competing interests: All authors have completed the ICMJE uniform disclosure form at www.icmje.org/coi_disclosure.pdf and declare: no support from any organization for the submitted work; no financial relationships except as noted below with any organizations that might have an interest in the submitted work in the previous three years; no other relationships or activities that could appear to have influenced the submitted work.

Ethical approval: The University of Warwick Biomedical Science Research Ethics Committee approved the study.

Transparency: The lead author affirms that this manuscript is an honest, accurate and transparent account of the study being reported; that no important aspects of the study have been omitted; and that any discrepancies from the study as planned (and, if relevant) have been explained.

For peer review only

Data sharing: No additional data available.

Acknowledgement: Work as part of the evaluation of the Best Care, Anywhere: Integrating Primary Care in Coventry Programme, which was essentially a collaborative effort between the Coventry – Rugby GP Alliance, the University Hospital Coventry and Warwickshire and academic partners, the University of Warwick, Coventry University and University of Birmingham. We would like to take this opportunity to express our gratitude to all members of the Coventry – Rugby GP Alliance Steering Group for their thoughtful discussions with the research team regarding the multi-faceted nature of the challenges currently facing the primary and acute care sectors in the region. We also gratefully acknowledge the contributions of University Hospital Coventry and Warwickshire for their efforts in sharing hospital activity data with the research team which made this study possible. Dr Wendy Robertson, Dr Victor Adekanmbi, Dr David Jenkinson and Professor Aileen Clarke are partly funded by the NIHR CLAHRC West Midlands initiative. Dr Clare Walker holds an NIHR funded ACF post. This report presents independent research and the views expressed are those of the author(s) and not necessarily those of the NHS, the NIHR or the Department of Health.

For peer review only

For peer review only - http://bmjopen.bmj.com/site/about/guidelines.xhtml

References

1. Carret ML, Fassa AC, Domingues MR. Inappropriate use of emergency services: a systematic review of prevalence and associated factors. *Cadernos de saude publica* 2009;25(1):7-28. [published Online First: 2009/01/31]

2. Lowy A, Kohler B, Nicholl J. Attendance at accident and emergency departments: unnecessary or inappropriate? *Journal of public health medicine* 1994;16(2):134-40. [published Online First: 1994/06/01]

3. Murphy AW. 'Inappropriate' attenders at accident and emergency departments I: definition, incidence and reasons for attendance. *Family practice* 1998;15(1):23-32. [published Online First: 1998/04/04]

4. Driscoll PA, Vincent CA, Wilkinson M. The use of the accident and emergency department. *Archives of emergency medicine* 1987;4(2):77-82. [published Online First: 1987/06/01]

5. Martin A, Martin C, Martin PB, et al. 'Inappropriate' attendance at an accident and emergency department by adults registered in local general practices: how is it related to their use of primary care? *Journal of health services research & policy* 2002;7(3):160-5. doi: 10.1258/135581902760082463 [published Online First: 2002/08/13]

6. Derlet RW, Richards JR. Overcrowding in the nation's emergency departments: complex causes and disturbing effects. *Annals of emergency medicine* 2000;35(1):63-8. [published Online First: 1999/12/30]

7. Siddiqui S, Ogbeide DO. Utilization of emergency services in a community hospital. *Saudi medical journal* 2002;23(1):69-72. [published Online First: 2002/04/09]

8. Roland M, Abel G. Reducing emergency admissions: are we on the right track? *BMJ (Clinical research ed)* 2012;345:e6017. doi: 10.1136/bmj.e6017 [published Online First: 2012/09/20]

9. Iacobucci G. All emergency departments should include GP staff, say experts. *BMJ (Clinical research ed)* 2014;349:g4654. doi: 10.1136/bmj.g4654 [published Online First: 2014/07/18]

10. Khangura JK, Flodgren G, Perera R, et al. Primary care professionals providing non-urgent care in hospital emergency departments. *The Cochrane database of systematic reviews* 2012;11:Cd002097. doi: 10.1002/14651858.CD002097.pub3 [published Online First: 2012/11/16]

11. Kmietowicz Z. GP triage could reduce attendances at emergency departments, study finds. *BMJ (Clinical research ed)* 2014;349:g7525. doi: 10.1136/bmj.g7525 [published Online First: 2014/12/10]

12. Dale J, Green J, Reid F, et al. Primary care in the accident and emergency department: II. Comparison of general practitioners and hospital doctors. *BMJ (Clinical research ed)* 1995;311(7002):427-30. [published Online First: 1995/08/12]

For peer review only - http://bmjopen.bmj.com/site/about/guidelines.xhtml

13. Gibney D, Murphy AW, Barton D, et al. Randomized controlled trial of general practitioner versus usual medical care in a suburban accident and emergency department using an informal triage system. *The British journal of general practice : the journal of the Royal College of General Practitioners* 1999;49(438):43-4. [published Online First: 2000/01/06]

14. Murphy AW, Bury G, Plunkett PK, et al. Randomised controlled trial of general practitioner versus usual medical care in an urban accident and emergency department: process, outcome, and comparative cost. *BMJ (Clinical research ed)* 1996;312(7039):1135-42. [published Online First: 1996/05/04]

15. Austin PC. Assessing balance in measured baseline covariates when using many-to-one matching on the propensity-score. *Pharmacoepidemiology and drug safety* 2008;17(12):1218-25. doi: 10.1002/pds.1674 [published Online First: 2008/10/31]

16. Campbell MJ. What is propensity score modelling? *Emergency medicine journal : EMJ* 2017;34(3):129-31. doi: 10.1136/emermed-2016-206542 [published Online First: 2017/02/02]

17. Freemantle N, Marston L, Walters K, et al. Making inferences on treatment effects from real world data: propensity scores, confounding by indication, and other perils for the unwary in observational research. *BMJ (Clinical research ed)* 2013;347:f6409. doi: 10.1136/bmj.f6409 [published Online First: 2013/11/13]

18. Kitsios GD, Dahabreh IJ, Callahan S, et al. Can We Trust Observational Studies Using Propensity Scores in the Critical Care Literature? A Systematic Comparison With Randomized Clinical Trials. *Critical care medicine* 2015;43(9):1870-9. doi: 10.1097/ccm.0000000000001135 [published Online First: 2015/06/19]

19. Craig P, Cooper C, Gunnell D, et al. Using natural experiments to evaluate population health interventions: new Medical Research Council guidance. *J Epidemiol Community Health* 2012;66(12):1182-6. doi: 10.1136/jech-2011-200375

For peer review only

For peer review only - http://bmjopen.bmj.com/site/about/guidelines.xhtml

Table 1: Basic characteristics of the patients included in the analyses.

	Before Matching				After Matching			
	Regular Emergency Physicians (n=114, 608)	GP in A&E (n=5426)	p-values	Standardized difference	Regular Emergency Physicians (n=10,852)	GP in A&E (n=5426)	p-value	Standardized difference
**Age mean, SD**	36.6 (26.2)	37.2 (18.9)	0.126	0.550	37.03 (19.2)	37.2 (18.9)	0.755	0.099
**Male (%)**	56,860 (49.6)	2,488 (45.9)		-0.038	5001 (46.1)	2,488 (45.9)	0.794	-0.002
**Ethnicity (%)**			<0.001				0.271	
**White**	**91,760 (80.1)**	**3,794 (69.9)**		-0.101	7,684 (70.8)	3,794 (69.9)		-0.009
**Asian**	10,904 (9.5)	699 (12.9)		0.034	1,401 (12.9)	699 (12.9)		0.000
**Black**	4,023 (3.5)	294 (5.4)		0.019	545 (5.0)	294 (5.4)		0.004
**Mixed**	2,603 (2.3)	117 (2.2)		-0.011	187 (1.7)	117 (2.2)		0.004
**Others**	5,318(4.6)	522 (9.6)		0.050	1035 (9.5)	522 (9.6)		0.001
**Distance (%)**			<0.001				0.844	
**< 5 miles**	34,491 (30.1)	2,351 (43.3)		0.009	4711 (43.4)	2,351 (43.3)		0.000
**5 – 10 miles**	48,261 (42.1)	2,237 (41.1)		-0.008	4492 (41.4)	2,237 (41.1)		-0.002
**10 to 20 miles**	**24,721 (21.6)**	**504 (9.3)**		-0.123	1014 (9.3)	504 (9.3)		-0.001
**20 to 50 miles**	4,763 (4.2)	156 (2.8)		-0.013	272 (2.5)	156 (2.8)		0.003
**> 50 miles**	2,372 (2.1)	181 (3.3)		0.013	363 (3.3)	181 (3.3)		0.000
**Presenting Symptoms (%)**			<0.001	-0.099			0.131	0.008
**0**	**57,317 (50.0)**	**3,196 (58.9)**			6408 (59.0)	3,196 (58.9)		
**1**	52,090 (45.5)	2,030 (37.4)			4094 (37.7)	2,030 (37.4)		
**2**	4,686 (4.1)	185 (3.4)			336 (3.1)	185 (3.4)		
**3 or more**	515 (0.4)	15 (0.3)			14 (0.1)	15 (0.3)		

For peer review only - http://bmjopen.bmj.com/site/about/guidelines.xhtml

FIGURE LEGENDS

Figure 1: Summary characteristics

Figure 2: Process outcomes and A&E clinical indicators

For peer review only

ONLINE ONLY TABLES

eTable 1: Any Intervention

eTable 2: Any Investigation

eTable 3: Patients' disposal method

For peer review only - http://bmjopen.bmj.com/site/about/guidelines.xhtml

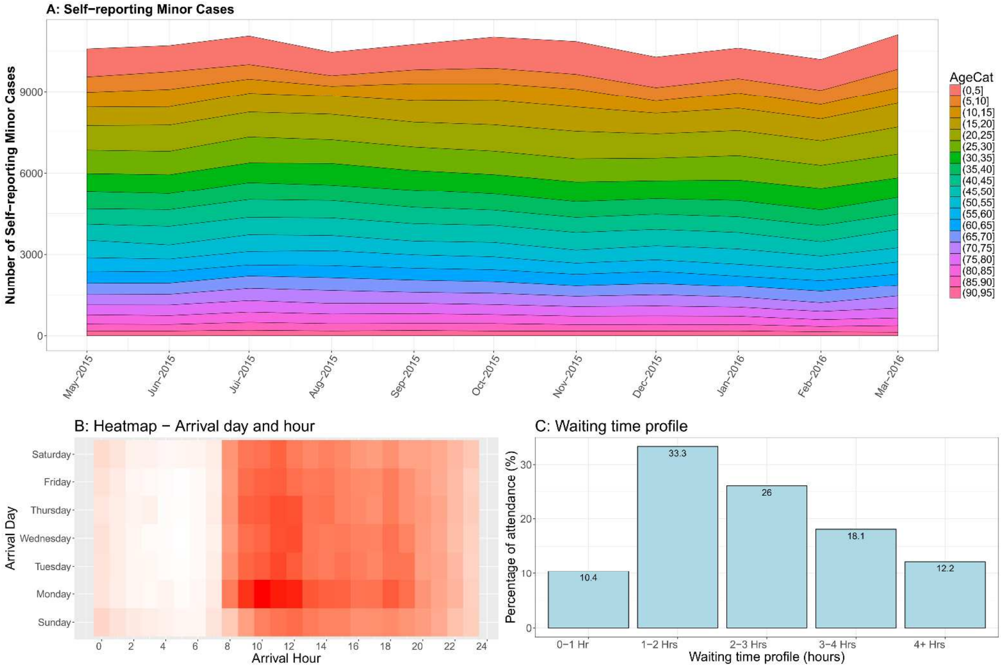

Figure 1: Summary characteristics

539x359mm (300 x 300 DPI)

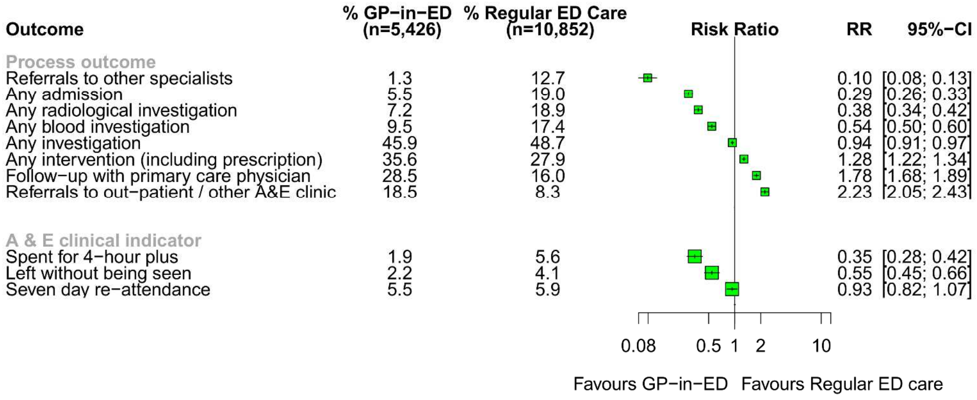

Figure 2: Process outcomes and A&E clinical indicators

976x400mm (96 x 96 DPI)

For peer review only - http://bmjopen.bmj.com/site/about/guidelines.xhtml

eTable 1: Any Intervention

	GP in ED		Regular Emergency Physician	
	No.	Col %	No.	Col %
Ametop	2	0.0	2	0.0
Arthropad	0	0.0	4	0.0
Backslab POP	0	0.0	12	0.1
Bandage/Support	3	0.0	43	0.3
Burns review	0	0.0	2	0.0
Chloramphenicol eye drops	16	0.2	40	0.2
Crutches	9	0.1	141	0.8
Digital nerve block	0	0.0	1	0.0
Digital splint	0	0.0	2	0.0
Dressing	33	0.3	350	2.1
Dressing/wound review	6	0.1	12	0.1
Eye irrigation	2	0.0	16	0.1
Falls prevention	0	0.0	1	0.0
Gastrostomy Tube Replacement	0	0.0	2	0.0
Guidance - verbal	4241	39.9	6678	39.3
Guidance - written	96	0.9	551	3.2
Guidance / Advice only	23	0.2	109	0.6
Infusion fluids	0	0.0	4	0.0
Intravenous cannula	68	0.6	133	0.8
Knee splint	1	0.0	4	0.0
Lavage/emesis/charcoal/eye irrigation	0	0.0	9	0.1
Manipulation	1	0.0	0	0.0
Medication - ear drops	10	0.1	7	0.0
Medication - eye drops	12	0.1	73	0.4
Medication - intra-nasal	2	0.0	2	0.0
Medication - oral	382	3.6	374	2.2
Medication - per rectum	29	0.3	41	0.2
Medication - topical skin cream	22	0.2	7	0.0
Medication administered	1	0.0	4	0.0
NSAID	184	1.7	223	1.3
Nasogastric Tube Replacement	0	0.0	3	0.0
Neighbour strapping	9	0.1	129	0.8
None (consider guidance/advice option)	11	0.1	37	0.2
Observation	3673	34.6	4534	26.7
Observation/electrocardiogram/pulse ox/head injury/trends	115	1.1	684	4.0
Oral Analgesia	476	4.5	722	4.2
Oral Rehydration Therapy	15	0.1	20	0.1
Oral antibiotics	408	3.8	376	2.2
Other (consider alternatives)	0	0.0	12	0.1
Prescription/medicines prepared to take away	663	6.2	690	4.1
Recording vital signs	0	0.0	10	0.1
Shortwave diathermy	1	0.0	0	0.0

For peer review only - http://bmjopen.bmj.com/site/about/guidelines.xhtml

	GP in ED		Regular Emergency Physician	
Sltra sound treatment	1	0.0	0	0.0
Sling/collar cuff/broad arm sling	7	0.1	90	0.5
Splint	3	0.0	11	0.1
Tetanus	2	0.0	3	0.0
Tetanus - human immunoglobulin	0	0.0	1	0.0
Tetanus - immune	0	0.0	1	0.0
Wound cleaning	34	0.3	388	2.3
Wound closure (excluding sutures)	0	0.0	8	0.0
Wound irrigation	1	0.0	56	0.3
Wrist splint	2	0.0	12	0.1
combined tetanus/diphtheria course	13	0.1	50	0.3
dressing minor wound/burn/eye	4	0.0	20	0.1
entonox	0	0.0	6	0.0
gait re-education	0	0.0	1	0.0
local anaesthetic	2	0.0	10	0.1
removal Plaster of Paris	0	0.0	1	0.0
removal of sutures/clips	1	0.0	13	0.1
steristrips	14	0.1	136	0.8
strapping	1	0.0	0	0.0
tetanus toxoid booster	0	0.0	3	0.0
wound glue	18	0.2	126	0.7
Total	10617	100.0	17000	100.0

For peer review only

For peer review only - http://bmjopen.bmj.com/site/about/guidelines.xhtml

eTable 2: Any Investigation

	GP in ED		Regular Emergency Physician	
	No.	Col %	No.	Col %
Arterial/capillary blood gas	12	0.1	132	0.6
BM Stix	1243	14.9	1259	6.2
Bacteriology	5	0.1	18	0.1
Biochemistry	19	0.2	119	0.6
Blood culture	10	0.1	86	0.4
CT (excludes GU contrast exam/tomography)	0	0.0	1	0.0
Cardiac enzymes	87	1.0	147	0.7
Clotting studies	106	1.3	594	2.9
Cross match blood/group and save serum for later cross match	4	0.0	38	0.2
D-Dimer	90	1.1	141	0.7
Dental Investigation	0	0.0	1	0.0
ESR	22	0.3	28	0.1
Electrocardiogram	748	9.0	788	3.9
Full blood count	583	7.0	2148	10.5
Glucose	63	0.8	291	1.4
Group & Save	24	0.3	206	1.0
Haematology	339	4.1	1644	8.0
Histology	0	0.0	3	0.0
INR	30	0.4	168	0.8
Immunology	2	0.0	1	0.0
Liver Function Test	490	5.9	1999	9.8
None	2855	34.2	4404	21.6
Orthoptic tests and computerised visual fields	0	0.0	14	0.1
Other	5	0.1	257	1.3
Other Biochemistry	0	0.0	1	0.0
Pregnancy test	165	2.0	413	2.0
Refraction	0	0.0	14	0.1
Serology	1	0.0	7	0.0
Tetanus	2	0.0	0	0.0
Toxicology	0	0.0	1	0.0
Urea & Electrolytes	574	6.9	2161	10.6
Urinalysis	414	5.0	890	4.4
Urine Culture	16	0.2	27	0.1
Wound Culture	0	0.0	5	0.0
X-ray plain film	433	5.2	2418	11.8
Total	8342	100.0	20424	100.0

For peer review only - http://bmjopen.bmj.com/site/about/guidelines.xhtml

eTable 3: Patients' disposal method

	**GP in ED**		**Regular Emergency Physician**	
	No.	Col %	No.	Col %
Clinic 9	0	0.0	916	8.4
Admitted to bed/became a LODGED PATIENT of the same Health Care Provider	301	5.5	2152	19.8
Died in Department	0	0.0	2	0.0
Discharged - did not require any follow up treatment	2370	43.7	4127	38.0
Discharged - follow up treatment to be provided by GP	1545	28.5	1733	16.0
Left Department before being treated	121	2.2	439	4.0
Left Department having refused treatment	4	0.1	41	0.4
Other	11	0.2	68	0.6
Referred to A&E Clinic	804	14.8	524	4.8
Referred to Fracture Clinic	22	0.4	301	2.8
Referred to other Out-Patient Clinic	198	3.6	374	3.4
Referred to other health care professional	46	0.8	161	1.5
Transferred to other Health Care Provider	4	0.1	14	0.1
Total	5426	100.0	10852	100.0

For peer review only

For peer review only - http://bmjopen.bmj.com/site/about/guidelines.xhtml

STROBE Statement—Checklist of items that should be included in reports of ***cross-sectional studies***

	Item No	Recommendation	Page
**Title and abstract**	1	(*a*) Indicate the study's design with a commonly used term in the title or the abstract	**1**
		(*b*) Provide in the abstract an informative and balanced summary of what was done and what was found	**2**
**Introduction**			
Background/rationale	2	Explain the scientific background and rationale for the investigation being reported	**3 - 4**
Objectives	3	State specific objectives, including any prespecified hypotheses	**4**
**Methods**			
Study design	4	Present key elements of study design early in the paper	**5**
Setting	5	Describe the setting, locations, and relevant dates, including periods of recruitment, exposure, follow-up, and data collection	**5-6**
Participants	6	(*a*) Give the eligibility criteria, and the sources and methods of selection of participants	**5-6**
Variables	7	Clearly define all outcomes, exposures, predictors, potential confounders, and effect modifiers. Give diagnostic criteria, if applicable	**7**
Data sources/ measurement	8*	For each variable of interest, give sources of data and details of methods of assessment (measurement). Describe comparability of assessment methods if there is more than one group	**5**
Bias	9	Describe any efforts to address potential sources of bias	**6**
Study size	10	Explain how the study size was arrived at	**6-7**
Quantitative variables	11	Explain how quantitative variables were handled in the analyses. If applicable, describe which groupings were chosen and why	**6-7**
Statistical methods	12	(*a*) Describe all statistical methods, including those used to control for confounding	**7-8**
		(*b*) Describe any methods used to examine subgroups and interactions	**7-8**
		(*c*) Explain how missing data were addressed	**7-8**
		(*d*) If applicable, describe analytical methods taking account of sampling strategy	**7-8**
		(*e*) Describe any sensitivity analyses	**7-8**
**Results**			
Participants	13*	(a) Report numbers of individuals at each stage of study—eg numbers potentially eligible, examined for eligibility, confirmed eligible, included in the study, completing follow-up, and analysed	**9**
		(b) Give reasons for non-participation at each stage	**9**
		(c) Consider use of a flow diagram	**9**
Descriptive data	14*	(a) Give characteristics of study participants (eg demographic, clinical, social) and information on exposures and potential confounders	**9**
		(b) Indicate number of participants with missing data for each variable of interest	**9**

For peer review only

For peer review only - http://bmjopen.bmj.com/site/about/guidelines.xhtml

Outcome data	15*	Report numbers of outcome events or summary measures	**10**
Main results	16	(*a*) Give unadjusted estimates and, if applicable, confounder-adjusted estimates and their precision (eg, 95% confidence interval). Make clear which confounders were adjusted for and why they were included	**10**
		(*b*) Report category boundaries when continuous variables were categorized	**10**
		(*c*) If relevant, consider translating estimates of relative risk into absolute risk for a meaningful time period	**10**
Other analyses	17	Report other analyses done—eg analyses of subgroups and interactions, and sensitivity analyses	**10**
**Discussion**			
Key results	18	Summarise key results with reference to study objectives	**11**
Limitations	19	Discuss limitations of the study, taking into account sources of potential bias or imprecision. Discuss both direction and magnitude of any potential bias	**12**
Interpretation	20	Give a cautious overall interpretation of results considering objectives, limitations, multiplicity of analyses, results from similar studies, and other relevant evidence	**11-13**
Generalisability	21	Discuss the generalisability (external validity) of the study results	**12-13**
**Other information**			
Funding	22	Give the source of funding and the role of the funders for the present study and, if applicable, for the original study on which the present article is based	**15**

For peer review only

BMJ Open

General practitioners providing non-urgent care in emergency department: a natural experiment

Journal:	*BMJ Open*
Manuscript ID	bmjopen-2017-019736.R1
Article Type:	Research
Date Submitted by the Author:	08-Jan-2018
Complete List of Authors:	Uthman, Olalekan; Warwick Medical School,<br>Walker, Clare; University of Warwick Warwick Medical School<br>Lahiri, Sudakshina; University of Warwick, Institute of Digital Healthcare, WMG<br>Jenkinson, David; University of Warwick, Warwick Medical School<br>Adekanmbi, Victor; University of Warwick Warwick Medical School<br>Robertson, Wendy; University of Warwick Warwick Medical School<br>Clarke, Aileen; University of Warwick, Division of Health Sciences
**Primary Subject Heading**:	Emergency medicine
Secondary Subject Heading:	General practice / Family practice
Keywords:	ACCIDENT & EMERGENCY MEDICINE, General practitioners, Four hour delay, Propensity score, natural experiment

SCHOLARONE™
Manuscripts

only

General practitioners providing non-urgent care in emergency department: a natural experiment

Olalekan A. Uthman[1], Clare Walker[1], Sudakshina Lahiri[2], David Jenkinson[1], Victor Adekanmbi[1], Wendy Robertson[1], Aileen Clarke[1]

[1]Division of Health Sciences, Warwick Medical School, The University of Warwick, Coventry, CV4 7AL, United Kingdom;

[2] Institute of Digital Healthcare, WMG, The University of Warwick, Coventry

Corresponding author: Prof. Aileen Clarke, Division of Health Sciences, Warwick Medical School, The University of Warwick, Coventry, CV4 7AL, United Kingdom;

For peer review only

For peer review only - http://bmjopen.bmj.com/site/about/guidelines.xhtml

ABSTRACT

Objective: To examine whether care provided by general practitioners (GPs) to non-urgent patients in the emergency department (ED) differs significantly from care provided by usual accident and emergency (A&E) staff in terms of process outcomes and A&E clinical quality indicators.

Design: Propensity score matched cohort study

Setting: GPs in A&E co-located within the University Hospitals Coventry and Warwickshire NHS Trust (UHCW) between May 2015 and March 2016.

Participants: Non-Urgent Attendances visits to the A&E department.

Main outcomes: Process outcomes (any investigation, any blood investigation, any radiological investigation, any intervention, admission and referrals) and A&E Clinical indicators (spent 4 hours plus, left without being seen, and seven-day re-attendance).

Results: A total of 5,426 patients seen by GPs in A&E were matched with 10,852 patients seen by emergency physicians (ratio 1:2). Compared with standard care in A&E, GPs in A&E significantly: admitted fewer patients (risk ratio [RR] 0.28, 95% confidence interval [CI] 0.25 to 0.31), referred fewer patients to other specialists (RR 0.31, 95% CI 0.24 to 0.40), ordered fewer radiological investigations (RR 0.38, 95% CI 0.34 to 0.42), ordered fewer blood tests (0.57, 95% CI 0.52 to 0.61), and ordered fewer investigations (0.93, 95% CI 0.90 to 0.96). However, they intervened more, offered more primary care follow up (RR 1.78, 95% CI 1.67 to 1.89), and referred more patients to out-patient and other A&E clinics (RR 2.29, 95% CI 2.10 to 2.49). Patients seen by GPs in A&E were on average less likely to spend four hours plus in A&E (RR 0.37, 95% CI 0.30 to 0.45) compared with standard care in A&E. There was no difference in re-attendance after seven days (RR 0.96, 95% CI 0.84 to 1.09).

Conclusion: GPs in A&E tended to manage self-reporting minor cases with fewer resources than standard care in A&E, without increasing re-attendance rates.

For peer review only - http://bmjopen.bmj.com/site/about/guidelines.xhtml

Strengths and limitations of this study

- Our results have important clinical implications as they inform optimal care for non-urgent care patients in A&E
- The main limitation of the study is the lack of random allocation and as such our findings may be prone to selection bias.
- We do not know the eventual health outcome, although re-attendance data are reassuring on this front.

For peer review only

For peer review only - http://bmjopen.bmj.com/site/about/guidelines.xhtml

BACKGROUND

Accident and Emergency departments (A&E) departments in the UK offer access 24 hours a day, 365 days a year and the immediate team usually includes paramedics, A&E nurses, diagnostic radiographers, A&E reception staff, porters, healthcare assistants and emergency medicine doctors[1]. Following arrival by ambulance or other self-determined mode of transport, the patient is triaged by a s qualified healthcare professional to ensure patients with the most serious conditions are seen first[1]. Depending on the presenting symptoms and signs, patients can either be treated, transferred to a nearby urgent care centre, minor injuries unit or referred to a GP on site or discharged[1]. Where treated, a patient is cared for by a team headed by a consultant in emergency medicine and the patients receive care from a suitably qualified member of the team.

A&E departments in the United Kingdom (UK) are facing a crisis in terms of overcrowding with long waiting times. Numerous factors have been suggested for this overcrowding in the use of A&E services, including ‘inappropriate’ use by patients with non-urgent conditions. A wide range (between 6.7% and 89%) of A&E visits has been classified as non-urgent depending on definition; and it is thought that many patients could have their care needs met in non-hospital including in General Practitioner (GP) settings [2-6]. Inappropriate use is thought to compromise A&E services for true emergencies [7 8]. And, it has been suggested, that non-urgent cases might be managed hastily or may not be linked in with the comprehensive and continuous care available in the primary care setting, thus leading to sub-optimal care [2].

For peer review only - http://bmjopen.bmj.com/site/about/guidelines.xhtml

Reducing inappropriate use of A&E has been an important area for policy makers for a long time[9-12], and experts have recently recommended that “All hospital emergency departments in the United Kingdom should include a primary care out-of-hours facility to help ease the ongoing pressure on the system” [13]. The “introduction of GPs may provide more comprehensive and cost- and resource-effective care for patients with non-urgent problems in the ED” it is thought [14]. It has also been suggested that GPs in A&E could reduce waiting times by seeing non-urgent patients promptly and freeing regular A&E physicians to see more urgent cases [15].

Previous studies have compared the effects of introducing GPs in A&E with standard care in A&E for managing non-urgent cases[14 16-18]. However, these studies have focused on process outcomes only. To our knowledge, no studies have examined the impact of GP in A&E on accident and emergency (A&E) clinical indicators. The present evaluation deals with a critical research gap highlighted by a previous Cochrane Review[14], described as the need for further studies of the effects of GP in A&E “on wait times, adverse effects, mortality and patient outcomes”. The main purpose of this study was therefore to examine whether care provided to non-urgent patients by GPs in the A&E differed significantly from care by usual A&E staff in terms of process outcomes and A&E clinical quality indicators.

For peer review only - http://bmjopen.bmj.com/site/about/guidelines.xhtml

METHODS

Study design, setting and source of data

We used anonymized individual level acute care activity data from the University Hospitals Coventry and Warwickshire NHS Trust (UHCW) between May 2015 and March 2016. We used the Urgent Care Project Board definition[19] to identify non-urgent attendances (self-reporting minor cases). These are unplanned visits to the A&E defined as those presenting with minor illness with a National Early Warning Score (NEWS) ≤ 1. The three relevant HRG codes are: VB08Z (Emergency Medicine, Category 2 investigation with Category 1 Treatment), VB09Z (Emergency Medicine, Category 1 investigation with Category 1 - 2 Treatment) and VB11Z (Emergency Medicine, No investigation with No Significant Treatment).

Intervention evaluated: GP in A&E scheme

The intervention of interest was whether the patients received care under the Secondary Care A&E team or by a GP in A&E. The GP in A&E scheme was set up by the Coventry and Rugby GP Alliance as part of the Prime Minister's GP Access Fund (PMAF) initiative. PMAF was a £50m Challenge Fund to support practices to try out new and innovative ways of delivering GP services and making services more accessible to patients.

The patients arriving at the A&E, were registered onto the Trust's clinical A&E system and triage was undertaken initially by the A&E triage nurse and by either an advanced nurse practitioner or a senior nurse, who also undertook an initial visual assessment of the

For peer review only - http://bmjopen.bmj.com/site/about/guidelines.xhtml

patient. Patients suitable for GP in A&E were identified and those deemed unsuitable were returned to existing A&E clinical pathways. Staff in the GP in A&E service also actively identified cases based on observation of patients in A&E, and/or based on information recorded in patient registration notes. Patients were not randomised. Once the decision to transfer to GP in A&E was made, the patient was moved onto the Trust's clinical A&E system by a receptionist and into the GP in A&E clinic. Patients suitable for the service were then diagnosed and treated by a GP in A&E. The patient's GP practice notes (accessed via the primary care shared care record) were available during the consultation. Patients who needed further tests or admission to hospital were referred to UHCW existing hospital services. Onward referral post discharge to a registered GP was via access to the primary care shared care record. The GP in A&E scheme operated seven days a week, for 12 hours per day at peak times.

Outcomes and covariates:

The outcomes of interest were:

1. Process outcomes: any investigations, any blood investigations, any radiological investigations, any intervention (including prescriptions) and admissions, discharges, referrals to outpatient clinics or other A&E clinics, referrals to other specialists, and follow-up treatment to be provided by GP in primary care setting.
2. A&E Clinical indicators: percentage of patients who spent 4 hours plus, who left without being seen, and seven-day re-attendance rates.

For peer review only - http://bmjopen.bmj.com/site/about/guidelines.xhtml

Confounding variables included in the analyses were patients' age, sex, ethnicity, number of procedures, distance to UHCW (partial post code), and number of presenting symptoms.

Statistical analyses

We examined the baseline characteristics of the patient cohort and estimated standardized differences for all variables before and after matching. A standardized difference of 10% or more is suggestive of imbalance [20]. We used propensity score[21] methods to account for all measured differences in baseline characteristics between patients seen by GP in A&E and standard care in A&E. The propensity score approach was used to control for all observed confounding factors that might influence assignment and outcome. We constructed a sample of patients balanced on covariates and risk factors (such as age, sex, ethnicity, number of procedures and number of presentations). We constructed the propensity scores using a logistic regression. We then matched each patient seen by GP in A&E to another similar standard care in A&E patients with the closest propensity score on a ratio of 1:2 using a nearest neighbour algorithm with no replacement. We calculated the average treatment effect on treated patients, which is a measure of the impact of GP in A&E on process outcomes and A&E clinical indicators. To test the robustness of our primary findings, we performed a series of sensitivity analyses. Firstly, we repeated the base case algorithm, nearest neighbour with replacement. Secondly, we also implemented another algorithm "exact" match with and without replacement. All data were analysed using R statistical software version 3.4.0. The null hypothesis was tested against a two-sided alternative hypothesis at significance level of 5%.

For peer review only - http://bmjopen.bmj.com/site/about/guidelines.xhtml

Patient involvement

The Collaborations for Leadership in Applied Health Research and Care (CLAHRC) West Midlands Patient and Public Involvement (PPI) advisors reviewed the study on a number of occasions. They were involved with the design of the research question and gave feedback at our advisory committee meeting. The research question was generated by the Coventry GP Alliance board who were the funders of the study. The results were discussed with PPI advisors and local authorities at a large stakeholder event. The results have been disseminated via our local authorities and shared through patient and public involvement initiatives.

For peer review only

For peer review only - http://bmjopen.bmj.com/site/about/guidelines.xhtml

RESULTS

Patients and Characteristics

A total of 170,154 patients attended UHCW A&E between May 2015 and March 2016 (Fig 1a). Our primary cohort consisted of 120,034 self-reporting minor case attendances in A&E at UHCW. Of every 10 attendees, 7 were self-reporting minor cases during this period. Attendances were relatively even throughout the week, with the highest attendances on a Monday (approximately 16%). Attendance was greatest on each day between 9-11am (Fig 1b). Most patients (70%) were dealt with, within 3 hours of attendance at A&E (Fig 1c).

Characteristics of the patients before and after matching are summarized in Table 1 (and supplementary eTable 1, eTable 2, and eTable 3). There were important baseline differences between patients seen by GPs in A&E and those seen in the normal pathway. GPs in A&E tended to see fewer white patients (69.9% versus 80.1%), and patients who lived closer to the hospital; 43.3% of GP in A&E patients lived within a 5 miles' radius of the A&E compared to 30.1% in standard care in A&E group. We successfully matched 5,426 patients seen by GPs in A&E with 10,852 standard care in A&E patients. After matching, absolute standardized differences were less than 10% for all variables used for the propensity score matching, suggesting an adequate match.

For peer review only

Process outcomes

Process outcomes and A&E clinical indicators are presented in Figure 2. GPs in A&E admitted significantly fewer patients with self-reporting minor cases (301/5426, 5.5%) than standard care in A&E (2168/10852, 20.1%; RR 0.28, 95% CI 0.25 to 0.31).

For peer review only - http://bmjopen.bmj.com/site/about/guidelines.xhtml

GPs in A&E referred fewer patients to other specialists (72/5426, 1.3%) than standard care in A&E (465/10852, 4.3%, Risk Ratio (RR) 0.31, 95% CI 0.24 to 0.40). GPs in A&E were significantly less likely than standard care in A&E to order radiological investigations (RR 0.38, 95% CI 0.34 to 0.42) and blood tests (RR 0.57, 95% CI 0.52 to 0.61) for patients. Also, on average, GPs in A&E ordered significantly but slightly fewer investigations overall, than standard care in A&E (RR 0.93, 95% CI 0.90 to 0.96). GPs in A&E intervened more (RR 1.29, 95% CI 1.23 to 1.35) and referred significantly more patients to out-patients and other A&E clinic (RR 2.29, 95% CI 2.10 to 2.49) than standard care in A&E. GPs in A&E also discharged more patients with the offer of significantly more primary care follow up than standard care in A&E(RR 1.78, 95% 1.67 to 1.89).

A&E Clinical Indicators

The proportion of patients (all self-reporting minor cases) who spent four hours plus in the A&E was statistically significantly lower for patients seen by the GPs in A&E (105/5426, 1.9%) than for those seen by standard care in A&E (573/10852, 5.3%; RR 0.37, 95% CI 0.30 to 0.45). Patients assigned to GP in A&E were less likely to leave without being seen (121/5426, 2.2%) compared with those assigned to standard care in A&E (427/10852, 3.9%; RR 0.57, 95% CI 0.46 to 0.69). The rate of seven-day re-attendance was similar among those patients seen by GP in A&E (296/5426, 5.5%) and emergency physicians (619/10852, 5.7%; RR 0.96, 95% CI 0.84 to 1.09).

For peer review only - http://bmjopen.bmj.com/site/about/guidelines.xhtml

For peer review only

Sensitivity Analyses

The results of the sensitivity analyses process outcomes and A&E clinical indicators are shown in **eFigure 1 and eFigure 1** respectively**.** The magnitudes and directions of the effect estimates were very similar to the primary analyses for both sensitivity analyses: the replacement method and an alternative matching algorithm "exact" with and without replacement. For example, the risk ratio for any admission ranged 0.27 to 0.29: nearest neighbour without replacement (RR 0.28, 95% CI 0.25 to 0.31), nearest neighbour replacement (RR 0.29, 95% CI 0.26 to 0.32), "exact" algorithm without replacement (RR 0.27, 95% CI 0.24 to 0.30), and "exact" algorithm replacement (RR 0.27, 95% CI 0.24 to 0.30).

For peer review only

For peer review only - http://bmjopen.bmj.com/site/about/guidelines.xhtml

DISCUSSION

Main findings

We found significant differences between, GPs in A&E and standard care in A&E. GPs in A&E referred fewer patients to other specialists, admitted fewer patients, ordered fewer radiological investigations, fewer blood tests, and fewer investigations overall and intervened more often. They offered more primary care follow up and referred more patients to out-patient and other A&E clinics. More importantly, on average, patients seen by GPs in A&E: were less likely to spend four hours plus in A&E and were no more likely to re-attend after seven days. The findings corroborate those of the previous three non-randomized studies[16-18] included in a Cochrane review[14].

The review involved more than 10,000 non-urgent cases from 52 A&E units, and provided evidence that GPs in A&E ordered fewer x-rays and admitted fewer patients compared with standard care in A&E. Our study also showed that GPs prescribed oral medication and to take out (TTO) medication more often, more oral antibiotics, more topical creams, and more ear drops, More TTOs may mean they are writing more repeat prescriptions or change existing prescriptions to optimise therapy. GPs prescribe less eye drops in general, slightly less rectal medication and nonsteroidal anti-inflammatory drugs (NSAIDs). Although they gave verbal guidance at similar rates as standard care in A&E.

As most of the A&E workload takes place from Monday to Friday and between 8am-10pm there is a case for extending coverage of GP in ED beyond these hours but not to 24 hours a day. Demand for primary care consultations at A&E locations reduce during the night and

For peer review only - http://bmjopen.bmj.com/site/about/guidelines.xhtml

alternative primary care services are available. NHS England's chief executive and the chief executive of NHS Improvement recently recommended that "Every hospital in England must have a "comprehensive" GP led triage system in emergency departments by October 2017 in a bid to avoid a repeat of the winter crisis that gripped the service this year"[22]. Two broad models of primary care services in A&E have been proposed [23]and these models are summarised in Box 1 [23]:

Box 1: Models of primary care services in A&E [23]

1. **Re-direct –** Patients present to the A&E and are sent to a primary care service:
    a) Adjacent out-of-hours service.
    b) Adjacent walk-in-centre.
    c) Adjacent primary care/community service.
    d) Advice only/self-care.
2. **Managing patients in the Emergency Department**
    a) **Gatekeeping in** A&E – Primary care service based at the front of A&E to manage patient entry to the A&E service.
    b) Primary care within A&E:
        i. GP working in A&E:
            1. Employed by primary care trust
            2. Employed by Acute Trust.
        ii. Other primary care clinician.
        iii. A&E clinician.

Our findings relate most closely to Model 1 -a redirect model with an adjacent primary care service.

There are several workforce implications, however. Many organisations are seeking to train and recruit GPs who can work in acute medicine settings in secondary and primary care centres with the expectation of developing a cadre of multi-skilled healthcare professionals[24]. This is in keeping with Model 2 with fully integrated primary care professionals in the A&E department. There is perhaps also a case for extending opportunities for secondary care A&E physicians to work in primary care during their

For peer review only - http://bmjopen.bmj.com/site/about/guidelines.xhtml

training to develop some of the GP skillset which has less reliance on tests and a greater experience and knowledge of which cases can safely be managed outside of the hospital[25]. However, recruitment and retention pressures in both specialities make this kind of collaborative training unlikely at present.

There is also the opportunity for the primary care approach to diffuse into the A&E – this is less likely in a self-contained, although co-located, unit than locations where primary and secondary care clinicians work in a more integrated fashion.

There may be a dilution of primary care skills and approaches over time for a GP fully integrated in A&E and their effectiveness will decrease over time as they become subsumed into the secondary care culture. However, if secondary care seek to recruit less experienced GPs to fill these roles, this may be more of a problem, as they may not have sufficient primary care experience as an independent practitioner to maintain their professional skills and identity in a secondary care environment. A GP in A&E requires an accommodation of a culture which is used to handling uncertainty with a culture which seeks to reduce uncertainty in diagnosis and treatment of patients.

Study limitations and strengths

The main limitation of the study is the lack of random allocation[26 27]. Our findings may be prone to selection bias since although we used a propensity score matching method to control for known baseline differences, it is possible that we have not controlled for

For peer review only - http://bmjopen.bmj.com/site/about/guidelines.xhtml

important confounders, such as severity of presentation. We did however adjust our analysis for number of presenting symptoms, number of procedures conducted and the number of tests ordered. In addition, we do not know the eventual health outcome, although re-attendance data are reassuring on this front.

Notwithstanding these limitations, the findings have some important policy and practice implications. Consistent with previous studies[16-18 28], these findings provide empirical evidence about GPs in A&E and A&E clinical indicators, supporting theories that "the introduction of GPs may provide more comprehensive and cost- and resource-effective care for patients with non-urgent problems in the A&E"[14] The 2016 and 2017 "extraordinary pressures" on A&E services across the country and schemes like this one will play a part in relieving this pressure. Our findings suggest that GPs are offering better continuity of care (perhaps more comprehensive care). We hypothesise that the fact that the GPs in A&E had access to primary care notes may be of importance here. Findings show that GPs made decisions more frequently about immediate onward referral to ambulatory clinics such as the urgent primary care clinic in UHCW or secondary care outpatient clinics. This is a more efficient use of resources avoiding both hospital admission and referral back to usual GP for them to make the referral. The removal of this unnecessary additional interaction reduces frustration for patients and doctors alike and should improve the patient experience.. In addition, the prescribing data we examined could mean that GPs in A&E are fulfilling requests for repeat medications more often than A&E staff with the added safety of access to the primary care record again reducing the need for additional communications with GPs.

For peer review only - http://bmjopen.bmj.com/site/about/guidelines.xhtml

We used data from one of the largest acute teaching hospitals in the UK. Our propensity score approach allowed us to reduce (though not eliminate) the possibility of confounding in relation to several relevant aspects of severity of presentation, as well as allowing us to balance a wide range of socio-demographic characteristics. The approach is consistent with Medical Research Council (MRC) guidelines for using natural experiments to evaluate population health interventions[29].

Conclusion

In this observational study of patients self-reporting with minor illness to the A&E department in one large teaching hospital, GPs in A&E made fewer referrals to other specialists, admitted fewer patients, ordered fewer radiological investigations, fewer blood tests, fewer investigations overall and intervened more often, compared to standard care in A&E. Overall they managed patients with fewer resources than standard care in A&E, without increasing re-attendance rates.

For peer review only

For peer review only - http://bmjopen.bmj.com/site/about/guidelines.xhtml

Contributors: OAU, CW, SL, DJ, WR, and AC were responsible for study concept and design. OAU and SL were responsible for acquisition of data. OAU, CW and AC drafted of the manuscript. OAU, SL, and VA performed the statistical analysis. All authors interpreted the data, critically revised the manuscript for important intellectual content and approved the final version for submission. All authors agree to be accountable for all aspects of the work. OAU and AC are guarantors.

Funding: Coventry – Rugby GP Alliance

Competing interests: All authors have completed the ICMJE uniform disclosure form at www.icmje.org/coi_disclosure.pdf and declare: no support from any organization for the submitted work; no financial relationships except as noted below with any organizations that might have an interest in the submitted work in the previous three years; no other relationships or activities that could appear to have influenced the submitted work.

Ethical approval: The University of Warwick Biomedical Science Research Ethics Committee approved the study.

Transparency: The lead author affirms that this manuscript is an honest, accurate and transparent account of the study being reported; that no important aspects of the study have been omitted; and that any discrepancies from the study as planned (and, if relevant) have been explained.

For peer review only

Data sharing: No additional data available.

Acknowledgement: Work as part of the evaluation of the Best Care, Anywhere: Integrating Primary Care in Coventry Programme, which was essentially a collaborative effort between the Coventry – Rugby GP Alliance, the University Hospital Coventry and Warwickshire and academic partners, the University of Warwick, Coventry University and University of Birmingham. We would like to take this opportunity to express our gratitude to all members of the Coventry – Rugby GP Alliance Steering Group for their thoughtful discussions with the research team regarding the multi-faceted nature of the challenges currently facing the primary and acute care sectors in the region. We also gratefully acknowledge the contributions of University Hospital Coventry and Warwickshire for their efforts in sharing hospital activity data with the research team which made this study possible. Dr Wendy Robertson, Dr Victor Adekanmbi, Dr David Jenkinson and Professor Aileen Clarke are partly funded by the NIHR CLAHRC West Midlands initiative. Dr Clare Walker holds an NIHR funded ACF post. Olalekan Uthman is supported by the National Institute of Health Research using Official Development Assistance (ODA) funding. This report presents independent research and the views expressed are those of the author(s) and not necessarily those of the NHS, the NIHR or the Department of Health.

For peer review only

References

1. NHS England. Urgent and emergency care services in England. [URL: https://www.nhs.uk/NHSEngland/AboutNHSservices/Emergencyandurgentcareservices/Pages/AE.aspx] Accessed 14 December 2017 2017 [

2. Carret ML, Fassa AC, Domingues MR. Inappropriate use of emergency services: a systematic review of prevalence and associated factors. *Cadernos de saude publica* 2009;25(1):7-28. [published Online First: 2009/01/31]

3. Lowy A, Kohler B, Nicholl J. Attendance at accident and emergency departments: unnecessary or inappropriate? *Journal of public health medicine* 1994;16(2):134-40. [published Online First: 1994/06/01]

4. Murphy AW. 'Inappropriate' attenders at accident and emergency departments I: definition, incidence and reasons for attendance. *Family practice* 1998;15(1):23-32. [published Online First: 1998/04/04]

5. Driscoll PA, Vincent CA, Wilkinson M. The use of the accident and emergency department. *Archives of emergency medicine* 1987;4(2):77-82. [published Online First: 1987/06/01]

6. Martin A, Martin C, Martin PB, et al. 'Inappropriate' attendance at an accident and emergency department by adults registered in local general practices: how is it related to their use of primary care? *Journal of health services research & policy* 2002;7(3):160-5. doi: 10.1258/135581902760082463 [published Online First: 2002/08/13]

7. Derlet RW, Richards JR. Overcrowding in the nation's emergency departments: complex causes and disturbing effects. *Annals of emergency medicine* 2000;35(1):63-8. [published Online First: 1999/12/30]

8. Siddiqui S, Ogbeide DO. Utilization of emergency services in a community hospital. *Saudi medical journal* 2002;23(1):69-72. [published Online First: 2002/04/09]

9. Roland M, Abel G. Reducing emergency admissions: are we on the right track? *BMJ (Clinical research ed)* 2012;345:e6017. doi: 10.1136/bmj.e6017 [published Online First: 2012/09/20]

10. Ismail SA, Gibbons DC, Gnani S. Reducing inappropriate accident and emergency department attendances: a systematic review of primary care service interventions. *The British journal of general practice : the journal of the Royal College of General Practitioners* 2013;63(617):e813-20. doi: 10.3399/bjgp13X675395 [published Online First: 2013/12/20]

11. Ramlakhan S, Mason S, O'Keeffe C, et al. Primary care services located with EDs: a review of effectiveness. *Emergency medicine journal : EMJ* 2016;33(7):495-503. doi: 10.1136/emermed-2015-204900 [published Online First: 2016/04/14]

For peer review only - http://bmjopen.bmj.com/site/about/guidelines.xhtml

12. Van den Heede K, Van de Voorde C. Interventions to reduce emergency department utilisation: A review of reviews. *Health policy (Amsterdam, Netherlands)* 2016;120(12):1337-49. doi: 10.1016/j.healthpol.2016.10.002 [published Online First: 2016/11/20]

13. Iacobucci G. All emergency departments should include GP staff, say experts. *BMJ (Clinical research ed)* 2014;349:g4654. doi: 10.1136/bmj.g4654 [published Online First: 2014/07/18]

14. Khangura JK, Flodgren G, Perera R, et al. Primary care professionals providing non-urgent care in hospital emergency departments. *The Cochrane database of systematic reviews* 2012;11:Cd002097. doi: 10.1002/14651858.CD002097.pub3 [published Online First: 2012/11/16]

15. Kmietowicz Z. GP triage could reduce attendances at emergency departments, study finds. *BMJ (Clinical research ed)* 2014;349:g7525. doi: 10.1136/bmj.g7525 [published Online First: 2014/12/10]

16. Dale J, Green J, Reid F, et al. Primary care in the accident and emergency department: II. Comparison of general practitioners and hospital doctors. *BMJ (Clinical research ed)* 1995;311(7002):427-30. [published Online First: 1995/08/12]

17. Gibney D, Murphy AW, Barton D, et al. Randomized controlled trial of general practitioner versus usual medical care in a suburban accident and emergency department using an informal triage system. *The British journal of general practice : the journal of the Royal College of General Practitioners* 1999;49(438):43-4. [published Online First: 2000/01/06]

18. Murphy AW, Bury G, Plunkett PK, et al. Randomised controlled trial of general practitioner versus usual medical care in an urban accident and emergency department: process, outcome, and comparative cost. *BMJ (Clinical research ed)* 1996;312(7039):1135-42. [published Online First: 1996/05/04]

19. (NECS); NNoECS, Capita;, England; UECACN. Transforming urgent and emergency care services in England: Urgent & Emergency Care Consolidated Channel Shift Model User Guide England: NHS England 2017.

20. Austin PC. Assessing balance in measured baseline covariates when using many-to-one matching on the propensity-score. *Pharmacoepidemiology and drug safety* 2008;17(12):1218-25. doi: 10.1002/pds.1674 [published Online First: 2008/10/31]

21. Campbell MJ. What is propensity score modelling? *Emergency medicine journal : EMJ* 2017;34(3):129-31. doi: 10.1136/emermed-2016-206542 [published Online First: 2017/02/02]

22. Iacobucci G. All emergency departments must have GP led triage by October. *BMJ (Clinical research ed)* 2017;356

For peer review only - http://bmjopen.bmj.com/site/about/guidelines.xhtml

23. Fisher JD, Salman B, Cooke MW. A rapid review exploring the interface between primary and emergency care in England. In: Carson D, Clay H, Stern R, eds. Primary Care and Emergency Departments: Primary Care Foundation 2010.

24. Moon M, Calderon A, Patel J, et al. The acute GP. *BMJ Careers* 2016

25. Nicol MF, McLauchlan CA. General practice: a secondment from emergency medicine--so what! *J Accid Emerg Med* 1998;15(4):218-9. [published Online First: 1998/07/29]

26. Freemantle N, Marston L, Walters K, et al. Making inferences on treatment effects from real world data: propensity scores, confounding by indication, and other perils for the unwary in observational research. *BMJ (Clinical research ed)* 2013;347:f6409. doi: 10.1136/bmj.f6409 [published Online First: 2013/11/13]

27. Kitsios GD, Dahabreh IJ, Callahan S, et al. Can We Trust Observational Studies Using Propensity Scores in the Critical Care Literature? A Systematic Comparison With Randomized Clinical Trials. *Critical care medicine* 2015;43(9):1870-9. doi: 10.1097/ccm.0000000000001135 [published Online First: 2015/06/19]

28. Smith L, Narang Y, Ibarz Pavon AB, et al. To GP or not to GP: a natural experiment in children triaged to see a GP in a tertiary paediatric emergency department (ED). *BMJ Quality & Safety* 2017

29. Craig P, Cooper C, Gunnell D, et al. Using natural experiments to evaluate population health interventions: new Medical Research Council guidance. *J Epidemiol Community Health* 2012;66(12):1182-6. doi: 10.1136/jech-2011-200375

For peer review only

For peer review only - http://bmjopen.bmj.com/site/about/guidelines.xhtml

Table 1: Basic characteristics of the patients included in the analyses.

	Before Matching				After Matching			
	Regular Emergency Physicians (n=114, 608)	GP in A&E (n=5426)	p-values	Standardized difference	Regular Emergency Physicians (n=10,852)	GP in A&E (n=5426)	p-value	Standardized difference
**Age mean, SD**	36.6 (26.2)	37.2 (18.9)	0.126	0.550	37.03 (19.2)	37.2 (18.9)	0.755	0.099
**Male (%)**	56,860 (49.6)	2,488 (45.9)		-0.038	5001 (46.1)	2,488 (45.9)	0.794	-0.002
**Ethnicity (%)**			<0.001				0.271	
**White**	**91,760 (80.1)**	**3,794 (69.9)**		-0.101	7,684 (70.8)	3,794 (69.9)		-0.009
**Asian**	10,904 (9.5)	699 (12.9)		0.034	1,401 (12.9)	699 (12.9)		0.000
**Black**	4,023 (3.5)	294 (5.4)		0.019	545 (5.0)	294 (5.4)		0.004
**Mixed**	2,603 (2.3)	117 (2.2)		-0.011	187 (1.7)	117 (2.2)		0.004
**Others**	5,318(4.6)	522 (9.6)		0.050	1035 (9.5)	522 (9.6)		0.001
**Distance (%)**			<0.001				0.844	
**< 5 miles**	34,491 (30.1)	2,351 (43.3)		0.009	4711 (43.4)	2,351 (43.3)		0.000
**5 – 10 miles**	48,261 (42.1)	2,237 (41.1)		-0.008	4492 (41.4)	2,237 (41.1)		-0.002
**10 to 20 miles**	**24,721 (21.6)**	**504 (9.3)**		-0.123	1014 (9.3)	504 (9.3)		-0.001
**20 to 50 miles**	4,763 (4.2)	156 (2.8)		-0.013	272 (2.5)	156 (2.8)		0.003
**> 50 miles**	2,372 (2.1)	181 (3.3)		0.013	363 (3.3)	181 (3.3)		0.000
**Presenting Symptoms (%)**			<0.001	-0.099			0.131	0.008
**0**	**57,317 (50.0)**	**3,196 (58.9)**			6408 (59.0)	3,196 (58.9)		
**1**	52,090 (45.5)	2,030 (37.4)			4094 (37.7)	2,030 (37.4)		
**2**	4,686 (4.1)	185 (3.4)			336 (3.1)	185 (3.4)		
**3 or more**	515 (0.4)	15 (0.3)			14 (0.1)	15 (0.3)		

For peer review only

For peer review only - http://bmjopen.bmj.com/site/about/guidelines.xhtml

FIGURE LEGENDS

Figure 1: Summary characteristics

Figure 2: Process outcomes and A&E clinical indicators

SUPPLEMENTARY MATERIALS

ONLINE ONLY TABLES

eTable 1: Any Intervention

eTable 2: Any Investigation

eTable 3: Patients' disposal method

ONLINE ONLY FIGURES

eFigure 1: Process outcomes, sensitivity analyses

eFigure 2: A&E clinical indicators, sensitivity analyses

For peer review only - http://bmjopen.bmj.com/site/about/guidelines.xhtml
For peer review only

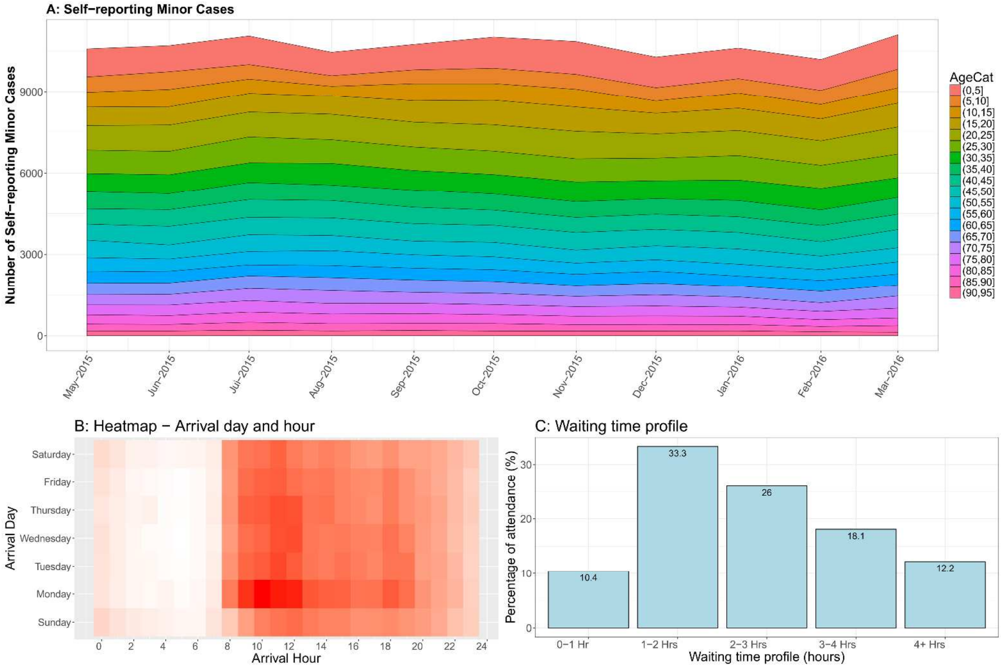

Figure 1: Summary characteristics

539x359mm (300 x 300 DPI)

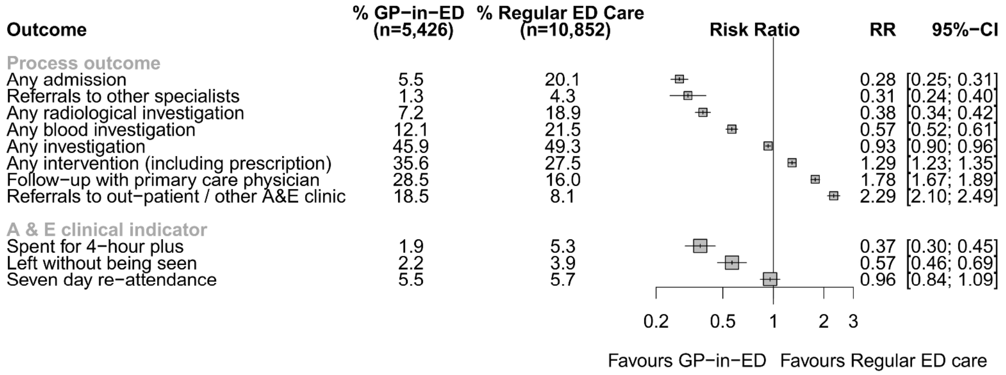

Figure 2

304x123mm (300 x 300 DPI)

eer review only

Contents

ONLINE ONLY TABLES 1

eTable 1: Any Intervention 1

eTable 2: Any Investigation 3

eTable 3: Patients' disposal method 4

ONLINE ONLY FIGURES 5

eFigure 1: Process outcomes, sensitivity analyses 5

eFigure 2: A&E clinical indicators, sensitivity analyses 6

For peer review only

ONLINE ONLY TABLES

eTable 1: Any Intervention

	GP in ED		Regular Emergency Physician	
	No.	Col %	No.	Col %
Ametop	2	0.0	2	0.0
Arthropad	0	0.0	4	0.0
Backslab POP	0	0.0	12	0.1
Bandage/Support	3	0.0	43	0.3
Burns review	0	0.0	2	0.0
Chloramphenicol eye drops	16	0.2	40	0.2
Crutches	9	0.1	141	0.8
Digital nerve block	0	0.0	1	0.0
Digital splint	0	0.0	2	0.0
Dressing	33	0.3	350	2.1
Dressing/wound review	6	0.1	12	0.1
Eye irrigation	2	0.0	16	0.1
Falls prevention	0	0.0	1	0.0
Gastrostomy Tube Replacement	0	0.0	2	0.0
Guidance - verbal	4241	39.9	6678	39.3
Guidance - written	96	0.9	551	3.2
Guidance / Advice only	23	0.2	109	0.6
Infusion fluids	0	0.0	4	0.0
Intravenous cannula	68	0.6	133	0.8
Knee splint	1	0.0	4	0.0
Lavage/emesis/charcoal/eye irrigation	0	0.0	9	0.1
Manipulation	1	0.0	0	0.0
Medication - ear drops	10	0.1	7	0.0
Medication - eye drops	12	0.1	73	0.4
Medication - intra-nasal	2	0.0	2	0.0
Medication - oral	382	3.6	374	2.2
Medication - per rectum	29	0.3	41	0.2
Medication - topical skin cream	22	0.2	7	0.0
Medication administered	1	0.0	4	0.0
NSAID	184	1.7	223	1.3
Nasogastric Tube Replacement	0	0.0	3	0.0
Neighbour strapping	9	0.1	129	0.8
None (consider guidance/advice option)	11	0.1	37	0.2
Observation	3673	34.6	4534	26.7
Observation/electrocardiogram/pulse ox/head injury/trends	115	1.1	684	4.0
Oral Analgesia	476	4.5	722	4.2
Oral Rehydration Therapy	15	0.1	20	0.1
Oral antibiotics	408	3.8	376	2.2
Other (consider alternatives)	0	0.0	12	0.1

For peer review only - http://bmjopen.bmj.com/site/about/guidelines.xhtml

	GP in ED		Regular Emergency Physician	
Prescription/medicines prepared to take away	663	6.2	690	4.1
Recording vital signs	0	0.0	10	0.1
Shortwave diathermy	1	0.0	0	0.0
Sltra sound treatment	1	0.0	0	0.0
Sling/collar cuff/broad arm sling	7	0.1	90	0.5
Splint	3	0.0	11	0.1
Tetanus	2	0.0	3	0.0
Tetanus - human immunoglobulin	0	0.0	1	0.0
Tetanus - immune	0	0.0	1	0.0
Wound cleaning	34	0.3	388	2.3
Wound closure (excluding sutures)	0	0.0	8	0.0
Wound irrigation	1	0.0	56	0.3
Wrist splint	2	0.0	12	0.1
combined tetanus/diphtheria course	13	0.1	50	0.3
dressing minor wound/burn/eye	4	0.0	20	0.1
entonox	0	0.0	6	0.0
gait re-education	0	0.0	1	0.0
local anaesthetic	2	0.0	10	0.1
removal Plaster of Paris	0	0.0	1	0.0
removal of sutures/clips	1	0.0	13	0.1
steristrips	14	0.1	136	0.8
strapping	1	0.0	0	0.0
tetanus toxoid booster	0	0.0	3	0.0
wound glue	18	0.2	126	0.7
Total	10617	100.0	17000	100.0

For peer review only

For peer review only - http://bmjopen.bmj.com/site/about/guidelines.xhtml

eTable 2: Any Investigation

	GP in ED		Regular Emergency Physician	
	No.	Col %	No.	Col %
Arterial/capillary blood gas	12	0.1	132	0.6
BM Stix	1243	14.9	1259	6.2
Bacteriology	5	0.1	18	0.1
Biochemistry	19	0.2	119	0.6
Blood culture	10	0.1	86	0.4
CT (excludes GU contrast exam/tomography)	0	0.0	1	0.0
Cardiac enzymes	87	1.0	147	0.7
Clotting studies	106	1.3	594	2.9
Cross match blood/group and save serum for later cross match	4	0.0	38	0.2
D-Dimer	90	1.1	141	0.7
Dental Investigation	0	0.0	1	0.0
ESR	22	0.3	28	0.1
Electrocardiogram	748	9.0	788	3.9
Full blood count	583	7.0	2148	10.5
Glucose	63	0.8	291	1.4
Group & Save	24	0.3	206	1.0
Haematology	339	4.1	1644	8.0
Histology	0	0.0	3	0.0
INR	30	0.4	168	0.8
Immunology	2	0.0	1	0.0
Liver Function Test	490	5.9	1999	9.8
None	2855	34.2	4404	21.6
Orthoptic tests and computerised visual fields	0	0.0	14	0.1
Other	5	0.1	257	1.3
Other Biochemistry	0	0.0	1	0.0
Pregnancy test	165	2.0	413	2.0
Refraction	0	0.0	14	0.1
Serology	1	0.0	7	0.0
Tetanus	2	0.0	0	0.0
Toxicology	0	0.0	1	0.0
Urea & Electrolytes	574	6.9	2161	10.6
Urinalysis	414	5.0	890	4.4
Urine Culture	16	0.2	27	0.1
Wound Culture	0	0.0	5	0.0
X-ray plain film	433	5.2	2418	11.8
Total	8342	100.0	20424	100.0

For peer review only - http://bmjopen.bmj.com/site/about/guidelines.xhtml

eTable 3: Patients' disposal method

	GP in ED		Regular Emergency Physician	
	No.	Col %	No.	Col %
Clinic 9	0	0.0	916	8.4
Admitted to bed/became a LODGED PATIENT of the same Health Care Provider	301	5.5	2152	19.8
Died in Department	0	0.0	2	0.0
Discharged - did not require any follow up treatment	2370	43.7	4127	38.0
Discharged - follow up treatment to be provided by GP	1545	28.5	1733	16.0
Left Department before being treated	121	2.2	439	4.0
Left Department having refused treatment	4	0.1	41	0.4
Other	11	0.2	68	0.6
Referred to A&E Clinic	804	14.8	524	4.8
Referred to Fracture Clinic	22	0.4	301	2.8
Referred to other Out-Patient Clinic	198	3.6	374	3.4
Referred to other health care professional	46	0.8	161	1.5
Transferred to other Health Care Provider	4	0.1	14	0.1
Total	5426	100.0	10852	100.0

For peer review only

For peer review only - http://bmjopen.bmj.com/site/about/guidelines.xhtml

ONLINE ONLY FIGURES

eFigure 1: Process outcomes, sensitivity analyses

Outcome	% GP−in−ED (n=5,426)	% Regular ED Care (n=10,852)	RR	95%−CI
**Any admission**				
Nearest Neighbour, without replacement	5.5	20.1	0.28	[0.25; 0.31]
Nearest Neighbour, with replacement	5.5	19.3	0.29	[0.26; 0.32]
Exact algorithm, without replacement	5.5	20.6	0.27	[0.24; 0.30]
Exact algorithm, with replacement	5.5	20.6	0.27	[0.24; 0.30]
**Referrals to other specialists**				
Nearest Neighbour, without replacement	1.3	4.3	0.31	[0.24; 0.40]
Nearest Neighbour, with replacement	1.3	4.3	0.31	[0.24; 0.39]
Exact algorithm, without replacement	1.3	4.3	0.31	[0.25; 0.39]
Exact algorithm, with replacement	1.3	4.3	0.31	[0.25; 0.39]
**Any radiological investigation**				
Nearest Neighbour, without replacement	7.2	18.9	0.38	[0.34; 0.42]
Nearest Neighbour, with replacement	7.2	18.3	0.39	[0.35; 0.43]
Exact algorithm, without replacement	7.2	19.0	0.38	[0.34; 0.42]
Exact algorithm, with replacement	7.2	19.0	0.38	[0.34; 0.42]
**Any blood investigation**				
Nearest Neighbour, without replacement	12.1	21.5	0.57	[0.52; 0.61]
Nearest Neighbour, with replacement	12.1	21.1	0.58	[0.53; 0.62]
Exact algorithm, without replacement	12.1	19.8	0.61	[0.57; 0.66]
Exact algorithm, with replacement	12.1	19.8	0.61	[0.57; 0.66]
**Any investigation**				
Nearest Neighbour, without replacement	45.9	49.3	0.93	[0.90; 0.96]
Nearest Neighbour, with replacement	45.9	49.6	0.92	[0.89; 0.96]
Exact algorithm, without replacement	45.8	47.1	0.97	[0.94; 1.00]
Exact algorithm, with replacement	45.8	47.1	0.97	[0.94; 1.00]
**Any intervention (including prescription)**				
Nearest Neighbour, without replacement	35.6	27.5	1.29	[1.23; 1.35]
Nearest Neighbour, with replacement	35.6	28.2	1.26	[1.20; 1.32]
Exact algorithm, without replacement	35.5	28.6	1.24	[1.20; 1.29]
Exact algorithm, with replacement	35.5	28.6	1.24	[1.20; 1.29]
**Follow−up with primary care physician**				
Nearest Neighbour, without replacement	28.5	16.0	1.78	[1.67; 1.89]
Nearest Neighbour, with replacement	28.5	16.7	1.70	[1.60; 1.81]
Exact algorithm, without replacement	28.6	14.7	1.94	[1.85; 2.03]
Exact algorithm, with replacement	28.6	14.7	1.94	[1.85; 2.03]
**Referrals to out−patient / other A&E clinic**				
Nearest Neighbour, without replacement	18.5	8.1	2.29	[2.10; 2.49]
Nearest Neighbour, with replacement	18.5	8.1	2.27	[2.08; 2.47]
Exact algorithm, without replacement	18.6	7.1	2.61	[2.45; 2.77]
Exact algorithm, with replacement	18.6	7.1	2.61	[2.45; 2.77]

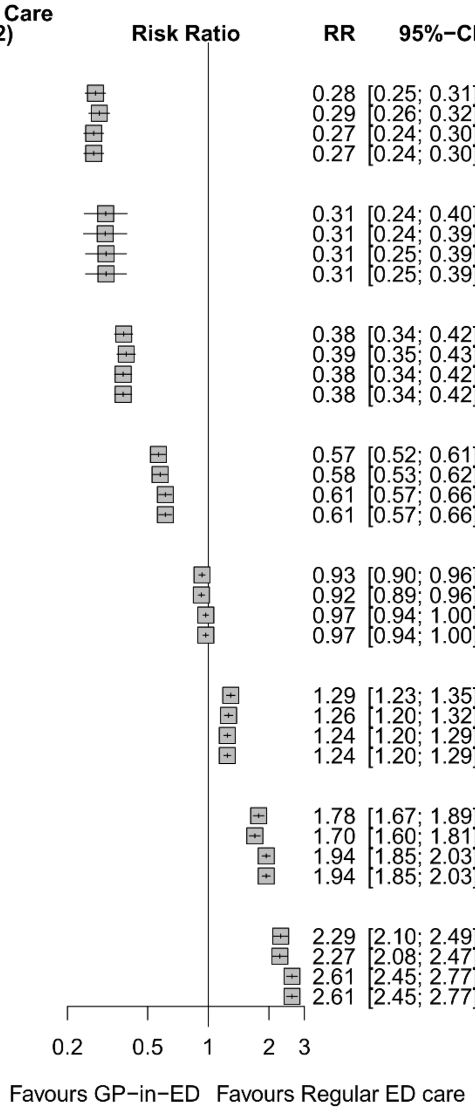

For peer review only - http://bmjopen.bmj.com/site/about/guidelines.xhtml

eFigure 2: A&E clinical indicators, sensitivity analyses

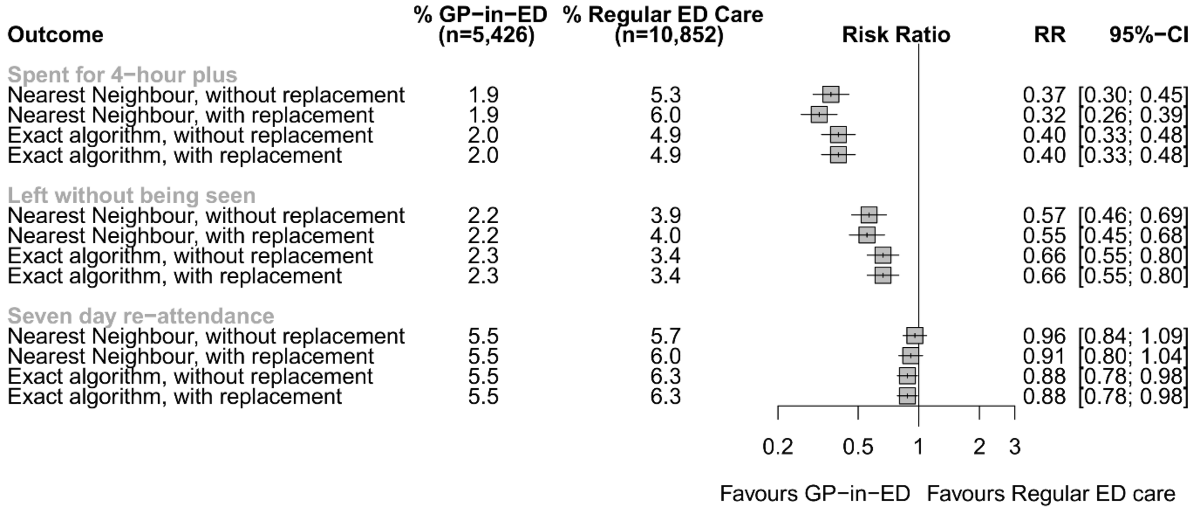

peer review only

For peer review only - http://bmjopen.bmj.com/site/about/guidelines.xhtml

STROBE Statement—Checklist of items that should be included in reports of ***cross-sectional studies***

	Item No	Recommendation	Page
**Title and abstract**	1	(*a*) Indicate the study's design with a commonly used term in the title or the abstract	**1**
		(*b*) Provide in the abstract an informative and balanced summary of what was done and what was found	**2**
**Introduction**			
Background/rationale	2	Explain the scientific background and rationale for the investigation being reported	**4-5**
Objectives	3	State specific objectives, including any prespecified hypotheses	**5**
**Methods**			
Study design	4	Present key elements of study design early in the paper	**6**
Setting	5	Describe the setting, locations, and relevant dates, including periods of recruitment, exposure, follow-up, and data collection	**6-7**
Participants	6	(*a*) Give the eligibility criteria, and the sources and methods of selection of participants	**6-7**
Variables	7	Clearly define all outcomes, exposures, predictors, potential confounders, and effect modifiers. Give diagnostic criteria, if applicable	**7-8**
Data sources/ measurement	8*	For each variable of interest, give sources of data and details of methods of assessment (measurement). Describe comparability of assessment methods if there is more than one group	**6**
Bias	9	Describe any efforts to address potential sources of bias	**7**
Study size	10	Explain how the study size was arrived at	**7-8**
Quantitative variables	11	Explain how quantitative variables were handled in the analyses. If applicable, describe which groupings were chosen and why	**7-8**
Statistical methods	12	(*a*) Describe all statistical methods, including those used to control for confounding	**8**
		(*b*) Describe any methods used to examine subgroups and interactions	**8**
		(*c*) Explain how missing data were addressed	**8**
		(*d*) If applicable, describe analytical methods taking account of sampling strategy	**8**
		(*e*) Describe any sensitivity analyses	**8**
**Results**			
Participants	13*	(a) Report numbers of individuals at each stage of study—eg numbers potentially eligible, examined for eligibility, confirmed eligible, included in the study, completing follow-up, and analysed	**10**
		(b) Give reasons for non-participation at each stage	**10**
		(c) Consider use of a flow diagram	**10**
Descriptive data	14*	(a) Give characteristics of study participants (eg demographic, clinical, social) and information on exposures and potential confounders	**10**
		(b) Indicate number of participants with missing data for each variable of interest	**10**

For peer review only

For peer review only - http://bmjopen.bmj.com/site/about/guidelines.xhtml

Outcome data	15*	Report numbers of outcome events or summary measures	**10**
Main results	16	(*a*) Give unadjusted estimates and, if applicable, confounder-adjusted estimates and their precision (eg, 95% confidence interval). Make clear which confounders were adjusted for and why they were included	**10-11**
		(*b*) Report category boundaries when continuous variables were categorized	**10-11**
		(*c*) If relevant, consider translating estimates of relative risk into absolute risk for a meaningful time period	**10-11**
Other analyses	17	Report other analyses done—eg analyses of subgroups and interactions, and sensitivity analyses	**12**
**Discussion**			
Key results	18	Summarise key results with reference to study objectives	**13-15**
Limitations	19	Discuss limitations of the study, taking into account sources of potential bias or imprecision. Discuss both direction and magnitude of any potential bias	**15-16**
Interpretation	20	Give a cautious overall interpretation of results considering objectives, limitations, multiplicity of analyses, results from similar studies, and other relevant evidence	**13-15**
Generalisability	21	Discuss the generalisability (external validity) of the study results	**13-15**
**Other information**			
Funding	22	Give the source of funding and the role of the funders for the present study and, if applicable, for the original study on which the present article is based	**18**

For peer review only

For peer review only - http://bmjopen.bmj.com/site/about/guidelines.xhtml

General practitioners providing non-urgent care in emergency department: a natural experiment

Journal:	*BMJ Open*
Manuscript ID	bmjopen-2017-019736.R2
Article Type:	Research
Date Submitted by the Author:	04-Apr-2018
Complete List of Authors:	Uthman, Olalekan; Warwick Medical School,<br>Walker, Clare; University of Warwick Warwick Medical School<br>Lahiri, Sudakshina; University of Warwick, Institute of Digital Healthcare, WMG<br>Jenkinson, David; University of Warwick, Warwick Medical School<br>Adekanmbi, Victor; University of Warwick Warwick Medical School<br>Robertson, Wendy; University of Warwick Warwick Medical School<br>Clarke, Aileen; University of Warwick, Division of Health Sciences
**Primary Subject Heading**:	Emergency medicine
Secondary Subject Heading:	General practice / Family practice
Keywords:	ACCIDENT & EMERGENCY MEDICINE, General practitioners, Four hour delay, Propensity score, natural experiment

SCHOLARONE™
Manuscripts

General practitioners providing non-urgent care in emergency department: a natural experiment

Olalekan A. Uthman[1], Clare Walker[1], Sudakshina Lahiri[2], David Jenkinson[1], Victor Adekanmbi[1], Wendy Robertson[1], Aileen Clarke[1]

[1]Division of Health Sciences, Warwick Medical School, The University of Warwick, Coventry, CV4 7AL, United Kingdom;

[2] Institute of Digital Healthcare, WMG, The University of Warwick, Coventry

Corresponding author: Prof. Aileen Clarke, Division of Health Sciences, Warwick Medical School, The University of Warwick, Coventry, CV4 7AL, United Kingdom;

Email: aileen.clarke@warwick.ac.uk

For peer review only - http://bmjopen.bmj.com/site/about/guidelines.xhtml

ABSTRACT

Objective: To examine whether care provided by general practitioners (GPs) to non-urgent patients in the emergency department (ED) differs significantly from care provided by usual accident and emergency (A&E) staff in terms of process outcomes and A&E clinical quality indicators.

Design: Propensity score matched cohort study

Setting: GPs in A&E co-located within the University Hospitals Coventry and Warwickshire NHS Trust (UHCW) between May 2015 and March 2016.

Participants: Non-Urgent Attendances visits to the A&E department.

Main outcomes: Process outcomes (any investigation, any blood investigation, any radiological investigation, any intervention, admission and referrals) and A&E Clinical indicators (spent 4 hours plus, left without being seen, and seven-day re-attendance).

Results: A total of 5,426 patients seen by GPs in A&E were matched with 10,852 patients seen by emergency physicians (ratio 1:2). Compared with standard care in A&E, GPs in A&E significantly: admitted fewer patients (risk ratio [RR] 0.28, 95% confidence interval [CI] 0.25 to 0.31), referred fewer patients to other specialists (RR 0.31, 95% CI 0.24 to 0.40), ordered fewer radiological investigations (RR 0.38, 95% CI 0.34 to 0.42), ordered fewer blood tests (0.57, 95% CI 0.52 to 0.61), and ordered fewer investigations (0.93, 95% CI 0.90 to 0.96). However, they intervened more, offered more primary care follow up (RR 1.78, 95% CI 1.67 to 1.89), and referred more patients to out-patient and other A&E clinics (RR 2.29, 95% CI 2.10 to 2.49). Patients seen by GPs in A&E were on average less likely to spend four hours plus in A&E (RR 0.37, 95% CI 0.30 to 0.45) compared with standard care in A&E. There was no difference in re-attendance after seven days (RR 0.96, 95% CI 0.84 to 1.09).

Conclusion: GPs in A&E tended to manage self-reporting minor cases with fewer resources than standard care in A&E, without increasing re-attendance rates.

For peer review only

For peer review only - http://bmjopen.bmj.com/site/about/guidelines.xhtml

Strengths and limitations of this study

- Our results have important clinical implications as they inform optimal care for non-urgent care patients in A&E
- The main limitation of the study is the lack of random allocation and as such our findings may be prone to selection bias.
- We do not know the eventual health outcome, although re-attendance data are reassuring on this front.

For peer review only

For peer review only - http://bmjopen.bmj.com/site/about/guidelines.xhtml

BACKGROUND

Accident and Emergency departments (A&E) departments in the UK offer access 24 hours a day, 365 days a year and the immediate team usually includes paramedics, A&E nurses, diagnostic radiographers, A&E reception staff, porters, healthcare assistants and emergency medicine doctors[1]. Following arrival by ambulance or other self-determined mode of transport, the patient is triaged by a s qualified healthcare professional to ensure patients with the most serious conditions are seen first[1]. Depending on the presenting symptoms and signs, patients can either be treated, transferred to a nearby urgent care centre, minor injuries unit or referred to a GP on site or discharged[1]. Where treated, a patient is cared for by a team headed by a consultant in emergency medicine and the patients receive care from a suitably qualified member of the team.

A&E departments in the United Kingdom (UK) are facing a crisis in terms of overcrowding with long waiting times. Numerous factors have been suggested for this overcrowding in the use of A&E services, including 'inappropriate' use by patients with non-urgent conditions. A wide range (between 6.7% and 89%) of A&E visits has been classified as non-urgent depending on definition; and it is thought that many patients could have their care needs met in non-hospital including in General Practitioner (GP) settings [2-6]. Inappropriate use is thought to compromise A&E services for true emergencies [7 8]. And, it has been suggested, that non-urgent cases might be managed hastily or may not be linked in with the comprehensive and continuous care available in the primary care setting, thus leading to sub-optimal care [2].

For peer review only - http://bmjopen.bmj.com/site/about/guidelines.xhtml

Reducing inappropriate use of A&E has been an important area for policy makers for a long time[9-12], and experts have recently recommended that "All hospital emergency departments in the United Kingdom should include a primary care out-of-hours facility to help ease the ongoing pressure on the system" [13]. The "introduction of GPs may provide more comprehensive and cost- and resource-effective care for patients with non-urgent problems in the ED" it is thought [14]. It has also been suggested that GPs in A&E could reduce waiting times by seeing non-urgent patients promptly and freeing regular A&E physicians to see more urgent cases [15].

Previous studies have compared the effects of introducing GPs in A&E with standard care in A&E for managing non-urgent cases[14 16-18]. However, these studies have focused on process outcomes only. To our knowledge, no studies have examined the impact of GP in A&E on accident and emergency (A&E) clinical indicators. The present evaluation deals with a critical research gap highlighted by a previous Cochrane Review[14], described as the need for further studies of the effects of GP in A&E "on wait times, adverse effects, mortality and patient outcomes". The main purpose of this study was therefore to examine whether care provided to non-urgent patients by GPs in the A&E differed significantly from care by usual A&E staff in terms of process outcomes and A&E clinical quality indicators.

For peer review only

For peer review only - http://bmjopen.bmj.com/site/about/guidelines.xhtml

METHODS

Study design, setting and source of data

We used anonymized individual level acute care activity data from the University Hospitals Coventry and Warwickshire NHS Trust (UHCW) between May 2015 and March 2016. We used the Urgent Care Project Board definition[19] to identify non-urgent attendances (self-reporting minor cases). These are unplanned visits to the A&E defined as those presenting with minor illness with a National Early Warning Score (NEWS) ≤ 1. The three relevant HRG codes are: VB08Z (Emergency Medicine, Category 2 investigation with Category 1 Treatment), VB09Z (Emergency Medicine, Category 1 investigation with Category 1 - 2 Treatment) and VB11Z (Emergency Medicine, No investigation with No Significant Treatment).

Intervention evaluated: GP in A&E scheme

The intervention of interest was whether the patients received care under the Secondary Care A&E team or by a GP in A&E. The GP in A&E scheme was set up by the Coventry and Rugby GP Alliance as part of the Prime Minister's GP Access Fund (PMAF) initiative. PMAF was a £50m Challenge Fund to support practices to try out new and innovative ways of delivering GP services and making services more accessible to patients.

The patients arriving at the A&E, were registered onto the Trust's clinical A&E system and triage was undertaken initially by the A&E triage nurse and by either an advanced nurse practitioner or a senior nurse, who also undertook an initial visual assessment of the

For peer review only - http://bmjopen.bmj.com/site/about/guidelines.xhtml

patient. Patients suitable for GP in A&E were identified and those deemed unsuitable were returned to existing A&E clinical pathways. Staff in the GP in A&E service also actively identified cases based on observation of patients in A&E, and/or based on information recorded in patient registration notes. Patients were not randomised. Once the decision to transfer to GP in A&E was made, the patient was moved onto the Trust's clinical A&E system by a receptionist and into the GP in A&E clinic. Patients suitable for the service were then diagnosed and treated by a GP in A&E. The patient's GP practice notes (accessed via the primary care shared care record) were available during the consultation. Patients who needed further tests or admission to hospital were referred to UHCW existing hospital services. Onward referral post discharge to a registered GP was via access to the primary care shared care record. The GP in A&E scheme operated seven days a week, for 12 hours per day at peak times.

Outcomes and covariates:

The outcomes of interest were:

1. Process outcomes: any investigations, any blood investigations, any radiological investigations, any intervention (including prescriptions) and admissions, discharges, referrals to outpatient clinics or other A&E clinics, referrals to other specialists, and follow-up treatment to be provided by GP in primary care setting.
2. A&E Clinical indicators: percentage of patients who spent 4 hours plus, who left without being seen, and seven-day re-attendance rates.

For peer review only - http://bmjopen.bmj.com/site/about/guidelines.xhtml

Confounding variables included in the analyses were patients' age, sex, ethnicity, number of procedures, distance to UHCW (partial post code), number of presenting symptoms, and arrival hour.

Statistical analyses

We examined the baseline characteristics of the patient cohort and estimated standardized differences for all variables before and after matching. A standardized difference of 10% or more is suggestive of imbalance [20]. We used propensity score[21] methods to account for all measured differences in baseline characteristics between patients seen by GP in A&E and standard care in A&E. The propensity score approach was used to control for all observed confounding factors that might influence assignment and outcome. We constructed a sample of patients balanced on covariates and risk factors (such as age, sex, ethnicity, number of procedures and number of presentations). We constructed the propensity scores using a logistic regression. We then matched each patient seen by GP in A&E to another similar standard care in A&E patients with the closest propensity score on a ratio of 1:2 using a nearest neighbour algorithm with no replacement. We calculated the average treatment effect on treated patients, which is a measure of the impact of GP in A&E on process outcomes and A&E clinical indicators. To test the robustness of our primary findings, we performed a series of sensitivity analyses. Firstly, we repeated the base case algorithm, nearest neighbour with replacement. Secondly, we also implemented another algorithm "exact" match with and without replacement. All data were analysed using R statistical software version 3.4.0. The null hypothesis was tested against a two-sided alternative hypothesis at significance level of 5%.

For peer review only - http://bmjopen.bmj.com/site/about/guidelines.xhtml

Patient involvement

The Collaborations for Leadership in Applied Health Research and Care (CLAHRC) West Midlands Patient and Public Involvement (PPI) advisors reviewed the study on a number of occasions. They were involved with the design of the research question and gave feedback at our advisory committee meeting. The research question was generated by the Coventry GP Alliance board who were the funders of the study. The results were discussed with PPI advisors and local authorities at a large stakeholder event. The results have been disseminated via our local authorities and shared through patient and public involvement initiatives.

For peer review only

For peer review only - http://bmjopen.bmj.com/site/about/guidelines.xhtml

RESULTS

Patients and Characteristics

A total of 170,154 patients attended UHCW A&E between May 2015 and March 2016 (Fig 1a). Our primary cohort consisted of 120,034 self-reporting minor case attendances in A&E at UHCW. Of every 10 attendees, 7 were self-reporting minor cases during this period. Attendances were relatively even throughout the week, with the highest attendances on a Monday (approximately 16%). Attendance was greatest on each day between 9-11am (Fig 1b). Most patients (70%) were dealt with, within 3 hours of attendance at A&E (Fig 1c).

Characteristics of the patients before and after matching are summarized in Table 1 (and supplementary eTable 1, eTable 2, and eTable 3). There were important baseline differences between patients seen by GPs in A&E and those seen in the normal pathway. GPs in A&E tended to see fewer white patients (69.9% versus 80.1%), and patients who lived closer to the hospital; 43.3% of GP in A&E patients lived within a 5 miles' radius of the A&E compared to 30.1% in standard care in A&E group. We successfully matched 5,426 patients seen by GPs in A&E with 10,852 standard care in A&E patients. After matching, absolute standardized differences were less than 10% for all variables used for the propensity score matching, suggesting an adequate match.

Process outcomes

Process outcomes and A&E clinical indicators are presented in Figure 2. GPs in A&E admitted significantly fewer patients with self-reporting minor cases (301/5426, 5.5%) than standard care in A&E (2168/10852, 20.1%; RR 0.28, 95% CI 0.25 to 0.31).

For peer review only - http://bmjopen.bmj.com/site/about/guidelines.xhtml

GPs in A&E referred fewer patients to other specialists (72/5426, 1.3%) than standard care in A&E (465/10852, 4.3%, Risk Ratio (RR) 0.31, 95% CI 0.24 to 0.40). GPs in A&E were significantly less likely than standard care in A&E to order radiological investigations (RR 0.38, 95% CI 0.34 to 0.42) and blood tests (RR 0.57, 95% CI 0.52 to 0.61) for patients. Also, on average, GPs in A&E ordered significantly but slightly fewer investigations overall, than standard care in A&E (RR 0.93, 95% CI 0.90 to 0.96). GPs in A&E intervened more (RR 1.29, 95% CI 1.23 to 1.35) and referred significantly more patients to out-patients and other A&E clinic (RR 2.29, 95% CI 2.10 to 2.49) than standard care in A&E. GPs in A&E also discharged more patients with the offer of significantly more primary care follow up than standard care in A&E(RR 1.78, 95% 1.67 to 1.89).

A&E Clinical Indicators

The proportion of patients (all self-reporting minor cases) who spent four hours plus in the A&E was statistically significantly lower for patients seen by the GPs in A&E (105/5426, 1.9%) than for those seen by standard care in A&E (573/10852, 5.3%; RR 0.37, 95% CI 0.30 to 0.45). Patients assigned to GP in A&E were less likely to leave without being seen (121/5426, 2.2%) compared with those assigned to standard care in A&E (427/10852, 3.9%; RR 0.57, 95% CI 0.46 to 0.69). The rate of seven-day re-attendance was similar among those patients seen by GP in A&E (296/5426, 5.5%) and emergency physicians (619/10852, 5.7%; RR 0.96, 95% CI 0.84 to 1.09).

For peer review only - http://bmjopen.bmj.com/site/about/guidelines.xhtml

Sensitivity Analyses

The results of the sensitivity analyses process outcomes and A&E clinical indicators are shown in **eFigure 1 and eFigure 2** respectively**.** The magnitudes and directions of the effect estimates were very similar to the primary analyses for both sensitivity analyses: the replacement method and an alternative matching algorithm “exact” with and without replacement. For example, the risk ratio for any admission ranged 0.27 to 0.29: nearest neighbour without replacement (RR 0.28, 95% CI 0.25 to 0.31), nearest neighbour replacement (RR 0.29, 95% CI 0.26 to 0.32), “exact” algorithm without replacement (RR 0.27, 95% CI 0.24 to 0.30), and “exact” algorithm replacement (RR 0.27, 95% CI 0.24 to 0.30).

For peer review only

For peer review only - http://bmjopen.bmj.com/site/about/guidelines.xhtml

DISCUSSION

Main findings

We found significant differences between, GPs in A&E and standard care in A&E. GPs in A&E referred fewer patients to other specialists, admitted fewer patients, ordered fewer radiological investigations, fewer blood tests, and fewer investigations overall and intervened more often. They offered more primary care follow up and referred more patients to out-patient and other A&E clinics. More importantly, on average, patients seen by GPs in A&E: were less likely to spend four hours plus in A&E and were no more likely to re-attend after seven days. The findings corroborate those of the previous three non-randomized studies[16-18] included in a Cochrane review[14].

The review involved more than 10,000 non-urgent cases from 52 A&E units, and provided evidence that GPs in A&E ordered fewer x-rays and admitted fewer patients compared with standard care in A&E. Our study also showed that GPs prescribed oral medication and to take out (TTO) medication more often, more oral antibiotics, more topical creams, and more ear drops, More TTOs may mean they are writing more repeat prescriptions or change existing prescriptions to optimise therapy. GPs prescribe less eye drops in general, slightly less rectal medication and nonsteroidal anti-inflammatory drugs (NSAIDs). Although they gave verbal guidance at similar rates as standard care in A&E.

As most of the A&E workload takes place from Monday to Friday and between 8am-10pm there is a case for extending coverage of GP in A&E beyond these hours but not to 24 hours a day. Demand for primary care consultations at A&E locations reduce during the night and

For peer review only - http://bmjopen.bmj.com/site/about/guidelines.xhtml

alternative primary care services are available. NHS England's chief executive and the chief executive of NHS Improvement recently recommended that "Every hospital in England must have a "comprehensive" GP led triage system in emergency departments by October 2017 in a bid to avoid a repeat of the winter crisis that gripped the service this year"[22]. Two broad models of primary care services in A&E have been proposed [23] and these models are summarised in Box 1 [23]:

Box 1: Models of primary care services in A&E [23]

1. **Re-direct –** Patients present to the A&E and are sent to a primary care service:
   a) Adjacent out-of-hours service.
   b) Adjacent walk-in-centre.
   c) Adjacent primary care/community service.
   d) Advice only/self-care.
2. **Managing patients in the Emergency Department**
   a) **Gatekeeping in** A&E – Primary care service based at the front of A&E to manage patient entry to the A&E service.
   b) Primary care within A&E:
      i. GP working in A&E:
         1. Employed by primary care trust
         2. Employed by Acute Trust.
      ii. Other primary care clinician.
      iii. A&E clinician.

Our findings relate most closely to Model 1 -a redirect model with an adjacent primary care service. Another recent systematic review examined the effectiveness of different models of delivering urgent care[24]. The review included 45 systematic reviews and 102 primary studies and found that the evidence on co-location of GP services with A&Es indicates that there is potential to improve care[24].

There are several workforce implications, however. Many organisations are seeking to train and recruit GPs who can work in acute medicine settings in secondary and primary care centres with the expectation of developing a cadre of multi-skilled healthcare

For peer review only - http://bmjopen.bmj.com/site/about/guidelines.xhtml

professionals[25]. This is in keeping with Model 2 with fully integrated primary care professionals in the A&E department. There is perhaps also a case for extending opportunities for secondary care A&E physicians to work in primary care during their training to develop some of the GP skillset which has less reliance on tests and a greater experience and knowledge of which cases can safely be managed outside of the hospital[26]. However, recruitment and retention pressures in both specialities make this kind of collaborative training unlikely at present.

There is also the opportunity for the primary care approach to diffuse into the A&E – this is less likely in a self-contained, although co-located, unit than locations where primary and secondary care clinicians work in a more integrated fashion.

There may be a dilution of primary care skills and approaches over time for a GP fully integrated in A&E and their effectiveness will decrease over time as they become subsumed into the secondary care culture. However, if secondary care seek to recruit less experienced GPs to fill these roles, this may be more of a problem, as they may not have sufficient primary care experience as an independent practitioner to maintain their professional skills and identity in a secondary care environment. A GP in A&E requires an accommodation of a culture which is used to handling uncertainty with a culture which seeks to reduce uncertainty in diagnosis and treatment of patients.

Study limitations and strengths

For peer review only - http://bmjopen.bmj.com/site/about/guidelines.xhtml

The main limitation of the study is the lack of random allocation[27 28]. Our findings may be prone to selection bias since although we used a propensity score matching method to control for known baseline differences, it is possible that we have not controlled for important confounders, such as severity of presentation. We did however adjust our analysis for number of presenting symptoms, number of procedures conducted and the number of tests ordered. In addition, we do not know the eventual health outcome, although re-attendance data are reassuring on this front.

Notwithstanding these limitations, the findings have some important policy and practice implications. Consistent with previous studies[16-18 29], these findings provide empirical evidence about GPs in A&E and A&E clinical indicators, supporting theories that "the introduction of GPs may provide more comprehensive and cost- and resource-effective care for patients with non-urgent problems in the A&E"[14] The 2016 and 2017 "extraordinary pressures" on A&E services across the country and schemes like this one will play a part in relieving this pressure. Our findings suggest that GPs are offering more comprehensive care. We hypothesise that the fact that the GPs in A&E had access to primary care notes may be of importance here. Findings show that GPs are more likely to make immediate onward referral to ambulatory clinics such as the urgent primary care clinic in UHCW or secondary care outpatient clinics. This is a more efficient use of resources avoiding both hospital admission and referral back to usual GP for them to make the referral. The removal of this unnecessary additional interaction reduces frustration for patients and doctors alike and should improve the patient experience.. In addition, the prescribing data we examined could mean that GPs in A&E are fulfilling requests for repeat medications more often than A&E

For peer review only - http://bmjopen.bmj.com/site/about/guidelines.xhtml

staff with the added safety of access to the primary care record again reducing the need for additional communications with GPs.

We used data from one of the largest acute teaching hospitals in the UK. Our propensity score approach allowed us to reduce (though not eliminate) the possibility of confounding in relation to several relevant aspects of severity of presentation, as well as allowing us to balance a wide range of socio-demographic characteristics. The approach is consistent with Medical Research Council (MRC) guidelines for using natural experiments to evaluate population health interventions[30].

Conclusion

In this observational study of patients self-reporting with minor illness to the A&E department in one large teaching hospital, GPs in A&E made fewer referrals to other specialists, admitted fewer patients, ordered fewer radiological investigations, fewer blood tests, fewer investigations overall and intervened more often, compared to standard care in A&E. Overall they managed patients with fewer resources than standard care in A&E, without increasing re-attendance rates.

For peer review only

For peer review only - http://bmjopen.bmj.com/site/about/guidelines.xhtml

Contributors: OAU, CW, SL, DJ, WR, and AC were responsible for study concept and design. OAU and SL were responsible for acquisition of data. OAU, CW and AC drafted of the manuscript. OAU, SL, and VA performed the statistical analysis. All authors interpreted the data, critically revised the manuscript for important intellectual content and approved the final version for submission. All authors agree to be accountable for all aspects of the work. OAU and AC are guarantors.

Funding: Coventry – Rugby GP Alliance

Competing interests: All authors have completed the ICMJE uniform disclosure form at www.icmje.org/coi_disclosure.pdf and declare: no support from any organization for the submitted work; no financial relationships except as noted below with any organizations that might have an interest in the submitted work in the previous three years; no other relationships or activities that could appear to have influenced the submitted work.

Ethical approval: The University of Warwick Biomedical Science Research Ethics Committee approved the study.

Transparency: The lead author affirms that this manuscript is an honest, accurate and transparent account of the study being reported; that no important aspects of the study have been omitted; and that any discrepancies from the study as planned (and, if relevant) have been explained.

For peer review only

For peer review only - http://bmjopen.bmj.com/site/about/guidelines.xhtml

Data sharing: No additional data available.

Acknowledgement: Work as part of the evaluation of the Best Care, Anywhere: Integrating Primary Care in Coventry Programme, which was essentially a collaborative effort between the Coventry – Rugby GP Alliance, the University Hospital Coventry and Warwickshire and academic partners, the University of Warwick, Coventry University and University of Birmingham. We would like to take this opportunity to express our gratitude to all members of the Coventry – Rugby GP Alliance Steering Group for their thoughtful discussions with the research team regarding the multi-faceted nature of the challenges currently facing the primary and acute care sectors in the region. We also gratefully acknowledge the contributions of University Hospital Coventry and Warwickshire for their efforts in sharing hospital activity data with the research team which made this study possible. Dr Wendy Robertson, Dr Victor Adekanmbi, Dr David Jenkinson and Professor Aileen Clarke are partly funded by the NIHR CLAHRC West Midlands initiative. Dr Clare Walker holds an NIHR funded ACF post. Olalekan Uthman is supported by the National Institute of Health Research using Official Development Assistance (ODA) funding. This report presents independent research and the views expressed are those of the author(s) and not necessarily those of the NHS, the NIHR or the Department of Health.

For peer review only - http://bmjopen.bmj.com/site/about/guidelines.xhtml

References

1. NHS England. Urgent and emergency care services in England. [URL: https://www.nhs.uk/NHSEngland/AboutNHSservices/Emergencyandurgentcareservices/Pages/AE.aspx] Accessed 14 December 2017 2017 [

2. Carret ML, Fassa AC, Domingues MR. Inappropriate use of emergency services: a systematic review of prevalence and associated factors. *Cadernos de saude publica* 2009;25(1):7-28. [published Online First: 2009/01/31]

3. Lowy A, Kohler B, Nicholl J. Attendance at accident and emergency departments: unnecessary or inappropriate? *Journal of public health medicine* 1994;16(2):134-40. [published Online First: 1994/06/01]

4. Murphy AW. 'Inappropriate' attenders at accident and emergency departments I: definition, incidence and reasons for attendance. *Family practice* 1998;15(1):23-32. [published Online First: 1998/04/04]

5. Driscoll PA, Vincent CA, Wilkinson M. The use of the accident and emergency department. *Archives of emergency medicine* 1987;4(2):77-82. [published Online First: 1987/06/01]

6. Martin A, Martin C, Martin PB, et al. 'Inappropriate' attendance at an accident and emergency department by adults registered in local general practices: how is it related to their use of primary care? *Journal of health services research & policy* 2002;7(3):160-5. doi: 10.1258/135581902760082463 [published Online First: 2002/08/13]

7. Derlet RW, Richards JR. Overcrowding in the nation's emergency departments: complex causes and disturbing effects. *Annals of emergency medicine* 2000;35(1):63-8. [published Online First: 1999/12/30]

8. Siddiqui S, Ogbeide DO. Utilization of emergency services in a community hospital. *Saudi medical journal* 2002;23(1):69-72. [published Online First: 2002/04/09]

9. Roland M, Abel G. Reducing emergency admissions: are we on the right track? *BMJ (Clinical research ed)* 2012;345:e6017. doi: 10.1136/bmj.e6017 [published Online First: 2012/09/20]

10. Ismail SA, Gibbons DC, Gnani S. Reducing inappropriate accident and emergency department attendances: a systematic review of primary care service interventions. *The British journal of general practice : the journal of the Royal College of General Practitioners* 2013;63(617):e813-20. doi: 10.3399/bjgp13X675395 [published Online First: 2013/12/20]

11. Ramlakhan S, Mason S, O'Keeffe C, et al. Primary care services located with EDs: a review of effectiveness. *Emergency medicine journal : EMJ* 2016;33(7):495-503. doi: 10.1136/emermed-2015-204900 [published Online First: 2016/04/14]

For peer review only - http://bmjopen.bmj.com/site/about/guidelines.xhtml

12. Van den Heede K, Van de Voorde C. Interventions to reduce emergency department utilisation: A review of reviews. *Health policy (Amsterdam, Netherlands)* 2016;120(12):1337-49. doi: 10.1016/j.healthpol.2016.10.002 [published Online First: 2016/11/20]

13. Iacobucci G. All emergency departments should include GP staff, say experts. *BMJ (Clinical research ed)* 2014;349:g4654. doi: 10.1136/bmj.g4654 [published Online First: 2014/07/18]

14. Khangura JK, Flodgren G, Perera R, et al. Primary care professionals providing non-urgent care in hospital emergency departments. *The Cochrane database of systematic reviews* 2012;11:Cd002097. doi: 10.1002/14651858.CD002097.pub3 [published Online First: 2012/11/16]

15. Kmietowicz Z. GP triage could reduce attendances at emergency departments, study finds. *BMJ (Clinical research ed)* 2014;349:g7525. doi: 10.1136/bmj.g7525 [published Online First: 2014/12/10]

16. Dale J, Green J, Reid F, et al. Primary care in the accident and emergency department: II. Comparison of general practitioners and hospital doctors. *BMJ (Clinical research ed)* 1995;311(7002):427-30. [published Online First: 1995/08/12]

17. Gibney D, Murphy AW, Barton D, et al. Randomized controlled trial of general practitioner versus usual medical care in a suburban accident and emergency department using an informal triage system. *The British journal of general practice : the journal of the Royal College of General Practitioners* 1999;49(438):43-4. [published Online First: 2000/01/06]

18. Murphy AW, Bury G, Plunkett PK, et al. Randomised controlled trial of general practitioner versus usual medical care in an urban accident and emergency department: process, outcome, and comparative cost. *BMJ (Clinical research ed)* 1996;312(7039):1135-42. [published Online First: 1996/05/04]

19. (NECS); NNoECS, Capita;, England; UECACN. Transforming urgent and emergency care services in England: Urgent & Emergency Care Consolidated Channel Shift Model User Guide England: NHS England 2017.

20. Austin PC. Assessing balance in measured baseline covariates when using many-to-one matching on the propensity-score. *Pharmacoepidemiology and drug safety* 2008;17(12):1218-25. doi: 10.1002/pds.1674 [published Online First: 2008/10/31]

21. Campbell MJ. What is propensity score modelling? *Emergency medicine journal : EMJ* 2017;34(3):129-31. doi: 10.1136/emermed-2016-206542 [published Online First: 2017/02/02]

22. Iacobucci G. All emergency departments must have GP led triage by October. *BMJ (Clinical research ed)* 2017;356

For peer review only

For peer review only - http://bmjopen.bmj.com/site/about/guidelines.xhtml

23. Fisher JD, Salman B, Cooke MW. A rapid review exploring the interface between primary and emergency care in England. In: Carson D, Clay H, Stern R, eds. Primary Care and Emergency Departments: Primary Care Foundation 2010.

24. Turner J, Coster J, Chambers D, et al. What evidence is there on the effectiveness of different models of delivering urgent care? A rapid review. *Health Services and Delivery Research* 2015 doi: 10.3310/hsdr03430

25. Moon M, Calderon A, Patel J, et al. The acute GP. *BMJ Careers* 2016

26. Nicol MF, McLauchlan CA. General practice: a secondment from emergency medicine--so what! *J Accid Emerg Med* 1998;15(4):218-9. [published Online First: 1998/07/29]

27. Freemantle N, Marston L, Walters K, et al. Making inferences on treatment effects from real world data: propensity scores, confounding by indication, and other perils for the unwary in observational research. *BMJ (Clinical research ed)* 2013;347:f6409. doi: 10.1136/bmj.f6409 [published Online First: 2013/11/13]

28. Kitsios GD, Dahabreh IJ, Callahan S, et al. Can We Trust Observational Studies Using Propensity Scores in the Critical Care Literature? A Systematic Comparison With Randomized Clinical Trials. *Critical care medicine* 2015;43(9):1870-9. doi: 10.1097/ccm.0000000000001135 [published Online First: 2015/06/19]

29. Smith L, Narang Y, Ibarz Pavon AB, et al. To GP or not to GP: a natural experiment in children triaged to see a GP in a tertiary paediatric emergency department (ED). *BMJ Quality & Safety* 2017

30. Craig P, Cooper C, Gunnell D, et al. Using natural experiments to evaluate population health interventions: new Medical Research Council guidance. *J Epidemiol Community Health* 2012;66(12):1182-6. doi: 10.1136/jech-2011-200375

For peer review only

For peer review only - http://bmjopen.bmj.com/site/about/guidelines.xhtml

Table 1: Basic characteristics of the patients included in the analyses.

	Before Matching				After Matching			
	Regular Emergency Physicians (n=114, 608)	GP in A&E (n=5426)	p-values	Standardized difference	Regular Emergency Physicians (n=10,852)	GP in A&E (n=5426)	p-value	Standardized difference
**Age mean, SD**	36.6 (26.2)	37.2 (18.9)	0.126	0.550	37.03 (19.2)	37.2 (18.9)	0.755	0.099
**Male (%)**	56,860 (49.6)	2,488 (45.9)		-0.038	5001 (46.1)	2,488 (45.9)	0.794	-0.002
**Ethnicity (%)**			<0.001				0.271	
**White**	**91,760 (80.1)**	**3,794 (69.9)**		-0.101	7,684 (70.8)	3,794 (69.9)		-0.009
**Asian**	10,904 (9.5)	699 (12.9)		0.034	1,401 (12.9)	699 (12.9)		0.000
**Black**	4,023 (3.5)	294 (5.4)		0.019	545 (5.0)	294 (5.4)		0.004
**Mixed**	2,603 (2.3)	117 (2.2)		-0.011	187 (1.7)	117 (2.2)		0.004
**Others**	5,318(4.6)	522 (9.6)		0.050	1035 (9.5)	522 (9.6)		0.001
**Distance (%)**			<0.001				0.844	
**< 5 miles**	34,491 (30.1)	2,351 (43.3)		0.009	4711 (43.4)	2,351 (43.3)		0.000
**5 – 10 miles**	48,261 (42.1)	2,237 (41.1)		-0.008	4492 (41.4)	2,237 (41.1)		-0.002
**10 to 20 miles**	**24,721 (21.6)**	**504 (9.3)**		-0.123	1014 (9.3)	504 (9.3)		-0.001
**20 to 50 miles**	4,763 (4.2)	156 (2.8)		-0.013	272 (2.5)	156 (2.8)		0.003
**> 50 miles**	2,372 (2.1)	181 (3.3)		0.013	363 (3.3)	181 (3.3)		0.000
**Presenting Symptoms (%)**			<0.001	-0.099			0.131	0.008
**0**	**57,317 (50.0)**	**3,196 (58.9)**			6408 (59.0)	3,196 (58.9)		
**1**	52,090 (45.5)	2,030 (37.4)			4094 (37.7)	2,030 (37.4)		
**2**	4,686 (4.1)	185 (3.4)			336 (3.1)	185 (3.4)		
**3 or more**	515 (0.4)	15 (0.3)			14 (0.1)	15 (0.3)		
**Arrival Hour mean, SD**	13.52 (5.51)	14.07 (3.52)	<0.001	0.5501	14.07 (4.62)	14.07 (3.52)	0.998	-0.000

For peer review only - http://bmjopen.bmj.com/site/about/guidelines.xhtml

FIGURE LEGENDS

Figure 1: Summary characteristics

Figure 2: Process outcomes and A&E clinical indicators

For peer review only

SUPPLEMENTARY MATERIALS

ONLINE ONLY TABLES

eTable 1: Any Intervention

eTable 2: Any Investigation

eTable 3: Patients' disposal method

ONLINE ONLY FIGURES

eFigure 1: Process outcomes, sensitivity analyses

eFigure 2: A&E clinical indicators, sensitivity analyses

For peer review only - http://bmjopen.bmj.com/site/about/guidelines.xhtml

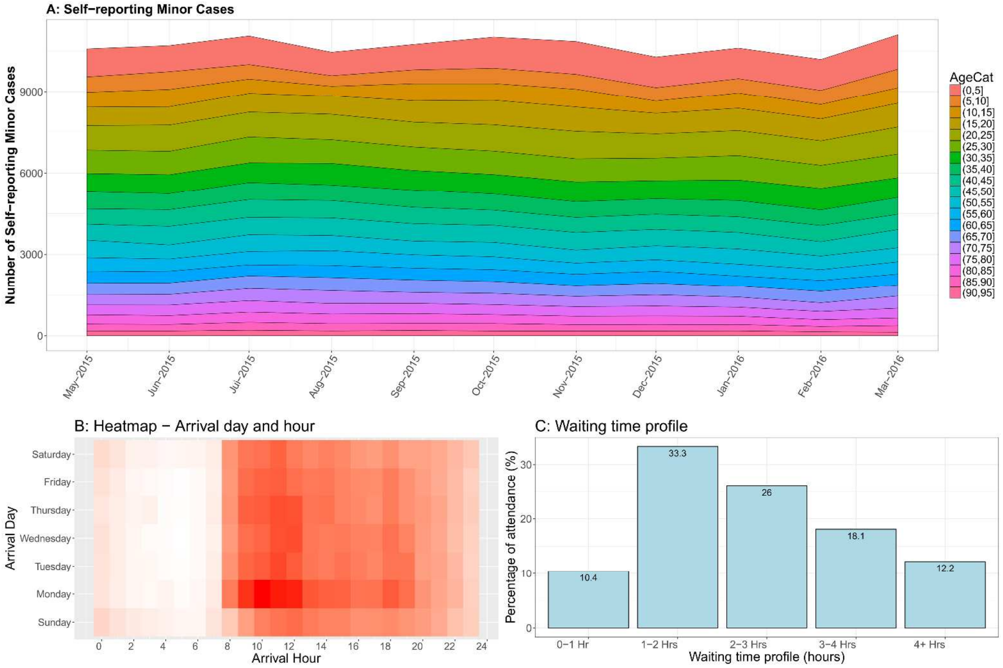

Figure 1

539x359mm (300 x 300 DPI)

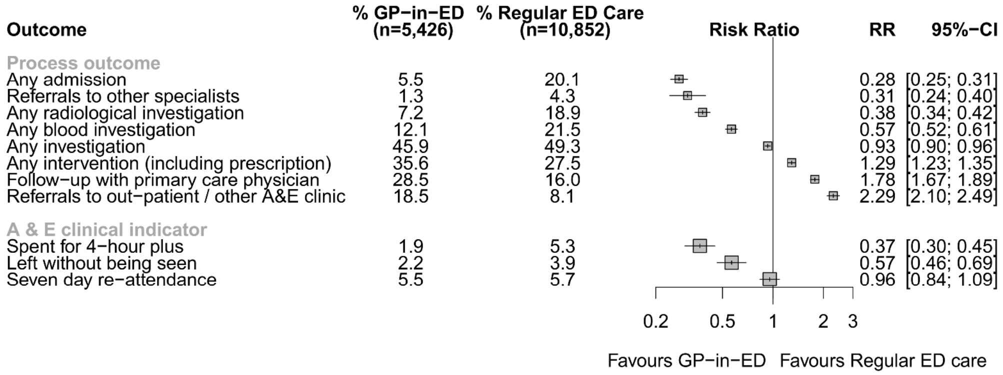

Figure 2

304x123mm (300 x 300 DPI)

For peer review only - http://bmjopen.bmj.com/site/about/guidelines.xhtml

Contents

ONLINE ONLY TABLES 1

eTable 1: Any Intervention 1

eTable 2: Any Investigation 3

eTable 3: Patients' disposal method 4

ONLINE ONLY FIGURES 5

eFigure 1: Process outcomes, sensitivity analyses 5

eFigure 2: A&E clinical indicators, sensitivity analyses 6

For peer review only

ONLINE ONLY TABLES

eTable 1: Any Intervention

	**GP in ED**		**Regular Emergency Physician**	
	No.	Col %	No.	Col %
Ametop	2	0.0	2	0.0
Arthropad	0	0.0	4	0.0
Backslab POP	0	0.0	12	0.1
Bandage/Support	3	0.0	43	0.3
Burns review	0	0.0	2	0.0
Chloramphenicol eye drops	16	0.2	40	0.2
Crutches	9	0.1	141	0.8
Digital nerve block	0	0.0	1	0.0
Digital splint	0	0.0	2	0.0
Dressing	33	0.3	350	2.1
Dressing/wound review	6	0.1	12	0.1
Eye irrigation	2	0.0	16	0.1
Falls prevention	0	0.0	1	0.0
Gastrostomy Tube Replacement	0	0.0	2	0.0
Guidance - verbal	4241	39.9	6678	39.3
Guidance - written	96	0.9	551	3.2
Guidance / Advice only	23	0.2	109	0.6
Infusion fluids	0	0.0	4	0.0
Intravenous cannula	68	0.6	133	0.8
Knee splint	1	0.0	4	0.0
Lavage/emesis/charcoal/eye irrigation	0	0.0	9	0.1
Manipulation	1	0.0	0	0.0
Medication - ear drops	10	0.1	7	0.0
Medication - eye drops	12	0.1	73	0.4
Medication - intra-nasal	2	0.0	2	0.0
Medication - oral	382	3.6	374	2.2
Medication - per rectum	29	0.3	41	0.2
Medication - topical skin cream	22	0.2	7	0.0
Medication administered	1	0.0	4	0.0
NSAID	184	1.7	223	1.3
Nasogastric Tube Replacement	0	0.0	3	0.0
Neighbour strapping	9	0.1	129	0.8
None (consider guidance/advice option)	11	0.1	37	0.2
Observation	3673	34.6	4534	26.7
Observation/electrocardiogram/pulse ox/head injury/trends	115	1.1	684	4.0
Oral Analgesia	476	4.5	722	4.2
Oral Rehydration Therapy	15	0.1	20	0.1
Oral antibiotics	408	3.8	376	2.2
Other (consider alternatives)	0	0.0	12	0.1

For peer review only - http://bmjopen.bmj.com/site/about/guidelines.xhtml

	GP in ED		Regular Emergency Physician	
Prescription/medicines prepared to take away	663	6.2	690	4.1
Recording vital signs	0	0.0	10	0.1
Shortwave diathermy	1	0.0	0	0.0
Sltra sound treatment	1	0.0	0	0.0
Sling/collar cuff/broad arm sling	7	0.1	90	0.5
Splint	3	0.0	11	0.1
Tetanus	2	0.0	3	0.0
Tetanus - human immunoglobulin	0	0.0	1	0.0
Tetanus - immune	0	0.0	1	0.0
Wound cleaning	34	0.3	388	2.3
Wound closure (excluding sutures)	0	0.0	8	0.0
Wound irrigation	1	0.0	56	0.3
Wrist splint	2	0.0	12	0.1
combined tetanus/diphtheria course	13	0.1	50	0.3
dressing minor wound/burn/eye	4	0.0	20	0.1
entonox	0	0.0	6	0.0
gait re-education	0	0.0	1	0.0
local anaesthetic	2	0.0	10	0.1
removal Plaster of Paris	0	0.0	1	0.0
removal of sutures/clips	1	0.0	13	0.1
steristrips	14	0.1	136	0.8
strapping	1	0.0	0	0.0
tetanus toxoid booster	0	0.0	3	0.0
wound glue	18	0.2	126	0.7
Total	10617	100.0	17000	100.0

For peer review only

eTable 2: Any Investigation

	GP in ED		Regular Emergency Physician	
	No.	Col %	No.	Col %
Arterial/capillary blood gas	12	0.1	132	0.6
BM Stix	1243	14.9	1259	6.2
Bacteriology	5	0.1	18	0.1
Biochemistry	19	0.2	119	0.6
Blood culture	10	0.1	86	0.4
CT (excludes GU contrast exam/tomography)	0	0.0	1	0.0
Cardiac enzymes	87	1.0	147	0.7
Clotting studies	106	1.3	594	2.9
Cross match blood/group and save serum for later cross match	4	0.0	38	0.2
D-Dimer	90	1.1	141	0.7
Dental Investigation	0	0.0	1	0.0
ESR	22	0.3	28	0.1
Electrocardiogram	748	9.0	788	3.9
Full blood count	583	7.0	2148	10.5
Glucose	63	0.8	291	1.4
Group & Save	24	0.3	206	1.0
Haematology	339	4.1	1644	8.0
Histology	0	0.0	3	0.0
INR	30	0.4	168	0.8
Immunology	2	0.0	1	0.0
Liver Function Test	490	5.9	1999	9.8
None	2855	34.2	4404	21.6
Orthoptic tests and computerised visual fields	0	0.0	14	0.1
Other	5	0.1	257	1.3
Other Biochemistry	0	0.0	1	0.0
Pregnancy test	165	2.0	413	2.0
Refraction	0	0.0	14	0.1
Serology	1	0.0	7	0.0
Tetanus	2	0.0	0	0.0
Toxicology	0	0.0	1	0.0
Urea & Electrolytes	574	6.9	2161	10.6
Urinalysis	414	5.0	890	4.4
Urine Culture	16	0.2	27	0.1
Wound Culture	0	0.0	5	0.0
X-ray plain film	433	5.2	2418	11.8
Total	8342	100.0	20424	100.0

For peer review only - http://bmjopen.bmj.com/site/about/guidelines.xhtml

eTable 3: Patients' disposal method

	GP in ED		Regular Emergency Physician	
	No.	Col %	No.	Col %
Clinic 9	0	0.0	916	8.4
Admitted to bed/became a LODGED PATIENT of the same Health Care Provider	301	5.5	2152	19.8
Died in Department	0	0.0	2	0.0
Discharged - did not require any follow up treatment	2370	43.7	4127	38.0
Discharged - follow up treatment to be provided by GP	1545	28.5	1733	16.0
Left Department before being treated	121	2.2	439	4.0
Left Department having refused treatment	4	0.1	41	0.4
Other	11	0.2	68	0.6
Referred to A&E Clinic	804	14.8	524	4.8
Referred to Fracture Clinic	22	0.4	301	2.8
Referred to other Out-Patient Clinic	198	3.6	374	3.4
Referred to other health care professional	46	0.8	161	1.5
Transferred to other Health Care Provider	4	0.1	14	0.1
Total	5426	100.0	10852	100.0

For peer review only

For peer review only - http://bmjopen.bmj.com/site/about/guidelines.xhtml

ONLINE ONLY FIGURES

eFigure 1: Process outcomes, sensitivity analyses

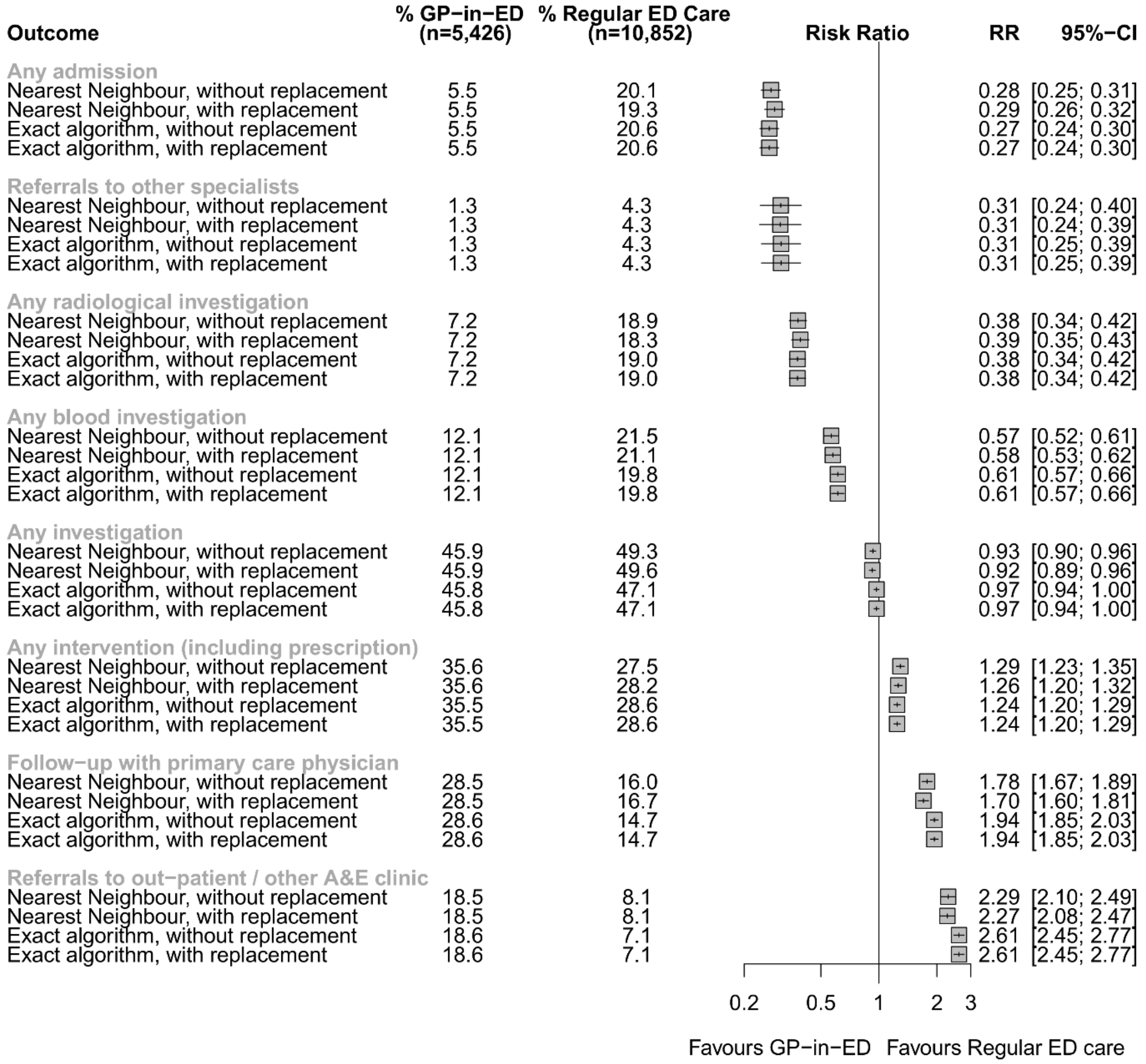

For peer review only - http://bmjopen.bmj.com/site/about/guidelines.xhtml

eFigure 2: A&E clinical indicators, sensitivity analyses

Outcome	% GP−in−ED (n=5,426)	% Regular ED Care (n=10,852)	RR	95%−CI
**Spent for 4−hour plus**				
Nearest Neighbour, without replacement	1.9	5.3	0.37	[0.30; 0.45]
Nearest Neighbour, with replacement	1.9	6.0	0.32	[0.26; 0.39]
Exact algorithm, without replacement	2.0	4.9	0.40	[0.33; 0.48]
Exact algorithm, with replacement	2.0	4.9	0.40	[0.33; 0.48]
**Left without being seen**				
Nearest Neighbour, without replacement	2.2	3.9	0.57	[0.46; 0.69]
Nearest Neighbour, with replacement	2.2	4.0	0.55	[0.45; 0.68]
Exact algorithm, without replacement	2.3	3.4	0.66	[0.55; 0.80]
Exact algorithm, with replacement	2.3	3.4	0.66	[0.55; 0.80]
**Seven day re−attendance**				
Nearest Neighbour, without replacement	5.5	5.7	0.96	[0.84; 1.09]
Nearest Neighbour, with replacement	5.5	6.0	0.91	[0.80; 1.04]
Exact algorithm, without replacement	5.5	6.3	0.88	[0.78; 0.98]
Exact algorithm, with replacement	5.5	6.3	0.88	[0.78; 0.98]

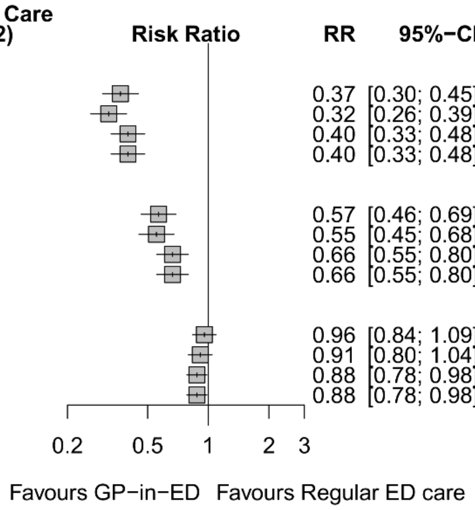

peer review only

STROBE Statement—Checklist of items that should be included in reports of ***cross-sectional studies***

	Item No	Recommendation	Page
**Title and abstract**	1	(*a*) Indicate the study's design with a commonly used term in the title or the abstract	**1**
		(*b*) Provide in the abstract an informative and balanced summary of what was done and what was found	**2**
**Introduction**			
Background/rationale	2	Explain the scientific background and rationale for the investigation being reported	**4-5**
Objectives	3	State specific objectives, including any prespecified hypotheses	**5**
**Methods**			
Study design	4	Present key elements of study design early in the paper	**6**
Setting	5	Describe the setting, locations, and relevant dates, including periods of recruitment, exposure, follow-up, and data collection	**6-7**
Participants	6	(*a*) Give the eligibility criteria, and the sources and methods of selection of participants	**6-7**
Variables	7	Clearly define all outcomes, exposures, predictors, potential confounders, and effect modifiers. Give diagnostic criteria, if applicable	**7-8**
Data sources/ measurement	8*	For each variable of interest, give sources of data and details of methods of assessment (measurement). Describe comparability of assessment methods if there is more than one group	**6**
Bias	9	Describe any efforts to address potential sources of bias	**7**
Study size	10	Explain how the study size was arrived at	**7-8**
Quantitative variables	11	Explain how quantitative variables were handled in the analyses. If applicable, describe which groupings were chosen and why	**7-8**
Statistical methods	12	(*a*) Describe all statistical methods, including those used to control for confounding	**8**
		(*b*) Describe any methods used to examine subgroups and interactions	**8**
		(*c*) Explain how missing data were addressed	**8**
		(*d*) If applicable, describe analytical methods taking account of sampling strategy	**8**
		(*e*) Describe any sensitivity analyses	**8**
**Results**			
Participants	13*	(a) Report numbers of individuals at each stage of study—eg numbers potentially eligible, examined for eligibility, confirmed eligible, included in the study, completing follow-up, and analysed	**10**
		(b) Give reasons for non-participation at each stage	**10**
		(c) Consider use of a flow diagram	**10**
Descriptive data	14*	(a) Give characteristics of study participants (eg demographic, clinical, social) and information on exposures and potential confounders	**10**
		(b) Indicate number of participants with missing data for each variable of interest	**10**

For peer review only - http://bmjopen.bmj.com/site/about/guidelines.xhtml
For peer review only

Outcome data	15*	Report numbers of outcome events or summary measures	**10**
Main results	16	(*a*) Give unadjusted estimates and, if applicable, confounder-adjusted estimates and their precision (eg, 95% confidence interval). Make clear which confounders were adjusted for and why they were included	**10-11**
		(*b*) Report category boundaries when continuous variables were categorized	**10-11**
		(*c*) If relevant, consider translating estimates of relative risk into absolute risk for a meaningful time period	**10-11**
Other analyses	17	Report other analyses done—eg analyses of subgroups and interactions, and sensitivity analyses	**12**
**Discussion**			
Key results	18	Summarise key results with reference to study objectives	**13-15**
Limitations	19	Discuss limitations of the study, taking into account sources of potential bias or imprecision. Discuss both direction and magnitude of any potential bias	**15-16**
Interpretation	20	Give a cautious overall interpretation of results considering objectives, limitations, multiplicity of analyses, results from similar studies, and other relevant evidence	**13-15**
Generalisability	21	Discuss the generalisability (external validity) of the study results	**13-15**
**Other information**			
Funding	22	Give the source of funding and the role of the funders for the present study and, if applicable, for the original study on which the present article is based	**18**

For peer review only
